# Oxidative Stress Mediates the Dual Regulatory Effects of Bovine Uterine ECM Remodeling Through the TGF-β1/Smad3 Pathway: Molecular Mechanisms of MMPs and COL-IV Imbalances

**DOI:** 10.3390/ani15131847

**Published:** 2025-06-23

**Authors:** Jiamei Tan, Zongjie Wang, Mingmao Yang, Ruihang Zhang, Zhongqiang Xue, Dong Zhou, Aihua Wang, Pengfei Lin, Yaping Jin

**Affiliations:** 1 Key Laboratory of Animal Biotechnology of the Ministry of Agriculture, College of Veterinary Medicine, Northwest A&F University, Yangling 712100, China; t924539104@163.com (J.T.); wangzongjie@nwafu.edu.cn (Z.W.); mingmao_yang@163.com (M.Y.); zhangruihang1230@163.com (R.Z.); askqiang@163.com (Z.X.); zhoudong1949@nwafu.edu.cn (D.Z.); aihuawang1966@163.com (A.W.); 2 Department of Clinical Veterinary Medicine, College of Veterinary Medicine, Northwest A&F University, Yangling 712100, China; 3 Department of Preventive Veterinary Medicine, College of Veterinary Medicine, Northwest A&F University, Yangling 712100, China

**Keywords:** oxidative stress, bovine endometritis, TGF-β1/Smad3 signaling pathway, MMPs, ECM remodeling

## Abstract

Bovine endometritis is a common endocrine and reproductive disorder in postpartum dairy cows, closely associated with elevated systemic oxidative stress. This disease can lead to delayed uterine involution, repeated breeding failure, and significant economic losses in the dairy industry. Studies suggest that oxidative stress may contribute to the pathological progression of endometritis by regulating ECM remodeling, but the specific molecular mechanisms remain unclear. ECM homeostasis relies on the coordinated action of matrix metalloproteinases (e.g., MMP2, MMP9) and collagen (e.g., type IV collagen, COL-IV), while the TGFβ1/Smad3 signaling pathway is implicated in ECM metabolic regulation. Therefore, elucidating the regulatory mechanisms of oxidative-stress-mediated TGFβ1/Smad3 signaling on ECM remodeling is crucial for understanding the pathogenesis of endometritis. This study investigates postpartum bovine uterine tissues, comparing inflammatory cytokines (IL-1β, IL-6, TNF-α) and oxidative-stress-related factors (GPx, SOD, CAT) between healthy and endometritis groups. Additionally, the differences in ECM-remodeling-associated proteins (MMP2, MMP9, COL-IV) and TGFβ1/Smad3 pathway activity are analyzed. To further validate the mechanisms, an oxidative stress model is established in vitro by treating bovine endometrial epithelial cells (bEECs) with 200 μM H_2_O_2_ for 4 h, followed by the valuation of the same indicators. Furthermore, gene silencing to downregulate Smad3 expression or inhibitor-mediated suppression of TGFβ1/Smad3 pathway activity is performed to observe their regulatory effects on MMP2, MMP9, and COL-IV. The results demonstrate that oxidative-stress-mediated endometritis significantly upregulates MMP2, MMP9, and the TGFβ1/Smad3 pathway activity, while suppressing COL-IV expression. Functional genetic experiments further reveal the dual regulatory role of the TGFβ1/Smad3 pathway in ECM remodeling: (1) pathway activation promotes MMP2/MMP9 expression, accelerating COL-IV degradation; (2) Smad3 positively regulates COL-IV synthesis. These findings provide a theoretical basis for targeting the TGFβ1/Smad3 pathway to mitigate the pathological progression of endometritis.

## Simple Summary

The remodeling of the extracellular matrix (ECM) is a critical process in postpartum uterine involution in dairy cows. Matrix metalloproteinases (MMPs), particularly MMP2 and MMP9, drive this dynamic process by degrading ECM components such as type IV collagen (COL-IV). Studies have shown that MMP2 and MMP9 expressions are significantly upregulated during uterine involution, facilitating the physiological remodeling of the uterine structure through the specific degradation of COL-IV, a major component of the basement membrane. However, the precise molecular mechanisms through which oxidative stress regulates ECM remodeling in bovine endometrial epithelial cells (bEECs) remain unclear. This study focuses on the TGFβ1/Smad3 signaling pathway to systematically investigate the regulatory effects of oxidative stress on ECM remodeling in bEECs. The experimental results reveal that postpartum oxidative stress and inflammatory responses exert dual regulatory effects on ECM remodeling by activating the TGFβ1/Smad3 pathway: (1) significantly upregulating MMP2/MMP9 expression to accelerate COL-IV degradation and (2) directly suppressing COL-IV synthesis, thereby disrupting the dynamic balance between ECM synthesis and degradation. This dual regulatory mechanism leads to ECM homeostasis disruption during uterine involution, ultimately causing incomplete uterine involution. These findings provide potential molecular targets for clinical intervention.

## 1. Introduction

Bovine endometritis predominantly occurs within 21 days postpartum, with its pathogenesis involving multiple contributing factors [1]. During parturition, the coordinated regulation of the levels of multiple hormones in the body induces contractions of the smooth muscles in the birth canal to facilitate fetal delivery [2]. This process triggers physiological stress in the dam and causes mucosal damage to the birth canal, creating entry points for pathogenic microorganisms such as Escherichia coli [3], Salmonella, and Staphylococcus aureus, thereby inducing endometrial inflammation [4]. Notably, a bidirectional vicious cycle exists between inflammatory responses and oxidative stress [5]: on the one hand, systemic oxidative stress postpartum activates inflammatory cascades [6]; on the other hand, the local inflammatory microenvironment exacerbates oxidative stress levels [7], forming a positive pathological feedback loop that complicates disease progression and prognosis.

The extracellular matrix (ECM), a non-cellular structural component in tissues and organs, not only provides physical support to cells but also plays critical roles in regulating tissue growth, inflammatory responses [8], injury repair [9], and homeostasis maintenance [10]. The ECM is primarily composed of five components: collagen, non-collagenous glycoproteins, elastin, proteoglycans, and glycosaminoglycans [11]. ECM remodeling refers to the dynamic synthesis, degradation, reorganization, and structural adjustment of these components, a process predominantly mediated by matrix metalloproteinases (MMPs) [12]. MMPs are zinc-dependent endopeptidases capable of degrading nearly all protein components in the ECM [13]. Among them, MMP2 and MMP9 act as key players by specifically degrading type IV collagen (COL-IV) to drive ECM remodeling [14]. Under oxidative stress, reactive oxygen species (ROS) levels significantly increase and regulate ECM remodeling through dual mechanisms: first, ROS directly activate MMP expression [15]; second, ROS inhibit the activity of the tissue inhibitors of metalloproteinases (TIMPs) via oxidative modification [16]. Additionally, inflammatory cytokines (e.g., IL-1β, IL-6, TNF-α) can bind to cell surface receptors to activate downstream signaling pathways, further upregulating MMP expression [17]. Periparturient dairy cows are prone to endometritis due to physiological immunosuppression and increased metabolic load, while abnormal ECM remodeling can delay uterine involution, severely impairing reproductive performance and production efficiency.

Transforming growth factor-β (TGF-β) is a multifunctional cytokine involved in embryonic development, immune regulation, tissue repair, and homeostasis maintenance [18]. Its signaling pathways can be classified into the canonical Smad-dependent pathway and the non-canonical Smad-independent pathway. In the canonical pathway, after the TGF-β1 ligand binds to the TGF-β receptor I (TGF-βRI) on the cell membrane, it recruits TGF-βRII to form a heterodimer, subsequently phosphorylating receptor-associated Smad proteins (Smad2/3) [19]. Phosphorylated Smad2/3 translocates to the nucleus, where it cooperates with transcription factors to regulate target gene expression [20]. The TGF-β1/Smad3 signaling pathway inhibitor SB-431542 can block this process by inhibiting the activities of ALK4, ALK5, and ALK7. It has been demonstrated to interfere with TGF-β signaling transduction, thereby ameliorating the epithelial–mesenchymal transition in human alveolar epithelial cells and reducing pulmonary fibrosis [21].

Recent studies indicate that the TGF-β1/Smad3 signaling pathway participates in ECM remodeling by regulating MMP expression [21]. For instance, TGF-β1 activates MMP2 expression via Smad2/3 nuclear translocation and upregulates MMP9 levels through the ERK1/2-JNK1/2-NF-κB cascade, thereby enhancing cell migration and invasion [22]. In a canine mammary tumor model, TGF-β/Smad pathway activation was significantly correlated with MMP9 overexpression, and MMP9 further amplified pathway activity via a negative feedback mechanism [23].

Furthermore, some studies indicate that upon activation of the TGFβ1/Smad3 signaling pathway, Smad3 translocates into the nucleus and can directly activate collagen transcription [24]. For instance, in primary human dermal fibroblasts, Smad3 binds to the COL1A2 promoter to enhance its expression [25]. In coronary artery disease, the downregulation of Smad3 expression was shown to abolish the stimulatory effect of TGFβ1 on COL4A1/COL4A2 [26]. These findings demonstrate that the TGFβ1/Smad3 signaling pathway activates the transcription of not only MMPs but also collagen proteins. This implies that in bovine endometritis, the TGFβ1/Smad3 signaling pathway may play a dual role in ECM remodeling.

This study aims to establish an in vitro oxidative stress model using hydrogen peroxide (H_2_O_2_)-induced bovine endometrial epithelial cells (bEECs) to simulate the pathological microenvironment of endometritis. By detecting the activity of the TGF-β/Smad3 signaling pathway and the dynamic expression of ECM-remodeling-related proteins (MMP2, MMP9, COL-IV), we systematically investigate the molecular mechanisms through which oxidative stress regulates ECM remodeling through the TGF-β/Smad3 pathway. This research is expected to provide a theoretical foundation for targeted interventions against bovine endometritis.

## 2. Materials and Methods

### 2.1. Sample Collection and Preparation

All experimental procedures used in this study complied with animal experimentation regulations and ethical requirements and were approved by the Laboratory Animal Ethics Committee of the College of Veterinary Medicine, Northwest A&F University (Approval No.: NWAFAC1027). The sample size was determined based on established conventions in similar studies [27].

All uterine samples in this study were obtained from Holstein cows. To exclude interference from other diseases, selected cows were free from mastitis, hoof diseases, or other health issues. Three cows diagnosed with endometritis and three healthy cows were slaughtered at an abattoir. Fresh uteri were preserved on ice and transported to the laboratory within two hours. The uterine cavity was fully exposed, rinsed with saline, and divided into two parts: one portion was immediately frozen in liquid nitrogen, while the other was fixed in 4% paraformaldehyde for subsequent experiments.

### 2.2. Cell Culture and Treatment

The bovine endometrial epithelial cell (bEEC) line stored in our laboratory was rapidly thawed in a 37 °C water bath and cultured in six-well plates. The culture medium consisted of 90% high-glucose Dulbecco’s Modified Eagle Medium (DMEM, BL304A, Biosharp, Guangzhou, China) and 10% fetal bovine serum (FBS, Z7186FBS-500, ZETA LIFE, San Francisco, CA, USA). Cells were maintained in a 37 °C incubator with 5% CO_2_ and saturated humidity. When cell density reached 70–80%, bEECs were treated with 0, 100, 200, 400, 600, 1000, 1200, or 1400 μM H_2_O_2_ for 4, 8, 12, or 24 h to assess cell viability and determine optimal treatment conditions.

Subsequently, cells were treated with 100 μM TGFβ receptor 1 inhibitor (TGFβRI1, sb431542) for 24–48 h before collection for downstream analyses.

### 2.3. RNA Isolation and RT-qPCR

Frozen uterine tissues were cut into small pieces, weighed, and ground into powder under liquid nitrogen. Total RNA was extracted using RNAiso Plus (Takara, Maebashi, Japan) following the manufacturer’s protocol. RNA concentration and purity were measured using a NanoDrop 2000 spectrophotometer (Thermo Scientific, Waltham, MA, USA) at 260/280 nm. Reverse transcription was performed using the Evo M-MLV Reverse Transcription Kit (Accurate Biology, AG11711, Accurate Biotechnology (Hunan) Co., Ltd, Hunan, China). RT-qPCR reactions were conducted as described in Table 1, and relative gene expression was quantified using SYBR Green Master Mix (CFX, Connect, Bio-Rad, Hercules, CA, USA) on a Bio-Rad CFX96 system. Primer sequences are listed in Table 2.

### 2.4. Protein Extraction and Western Blot

Frozen uterine tissues were homogenized in liquid nitrogen, and cells were scraped from six-well plates. Proteins were extracted using RIPA buffer containing 1% protease inhibitor cocktail. Protein concentration was determined via BCA assay (KGP902, Nanjing Keygen, Nanjing, China). Proteins were separated on 10% or 12% SDS-PAGE gels and transferred to PVDF membranes. Membranes were blocked with 10% skim milk in TBST (50 mM Tris, 150 mM NaCl, 0.1% Tween-20, pH 7.4) for 2 h, then incubated overnight at 4 °C with primary antibodies against TGF-β1 (sc-130348), Smad3 (AF1501), P-Smad3 (AF1759), MMP2 (66366-1-Ig), MMP9 (27306-1-AP), or COL-IV (30850-1-AP). After washing, membranes were incubated with HRP-conjugated secondary antibodies (1:5000, ZhongHuihecai, PB001 or PB002, Xi’an, China) for 1 h at room temperature. Protein bands were visualized using an enhanced chemiluminescence system (Tanon Biotech, Shanghai, China) and analyzed with ImageJ 1.47v software.

### 2.5. Translation

bEECs were cultured in a 6-well plate. When the cells reach 70–80% confluence, 2 μg of plasmid was transfected using TurboFect™ (Thermo Fisher Scientific, Waltham, MA, USA) according to the manufacturer’s instructions. The sequences of si-NC and si-Smad3 are listed in Table 3. For all transfection experiments, Si-NC was used as the negative control. Protein detection employed β-actin as the internal reference, and transfection efficiency was monitored based on the Smad3 protein detection results.

### 2.6. Immunohistochemistry (IHC)

Tissue sections were baked at 65 °C for 45 min, deparaffinized in xylene, and rehydrated through graded ethanol. Antigen retrieval was performed in 95 °C citrate buffer (pH 6.0) for 15 min. Endogenous peroxidase activity was quenched with 3% H_2_O_2_. Sections were blocked with 5% BSA, incubated with primary antibodies overnight at 4 °C, and then treated with HRP-conjugated secondary antibodies for 30 min. Color development was achieved using DAB substrate, followed by hematoxylin counterstaining. Sections were dehydrated, cleared in xylene, and mounted with neutral resin for microscopic observation. Positive results in immunohistochemistry were determined based on the intensity of brownish-yellow DAB staining.

### 2.7. Immunofluorescence (IF)

Cells grown on coverslips in 24-well plates were fixed with 4% paraformaldehyde at 4 °C overnight, permeabilized with 0.1% Triton X-100, and blocked with 1% BSA. Primary antibodies were applied overnight at 4 °C, followed by Alexa Fluor-conjugated secondary antibodies (1:500) and DAPI staining. Coverslips were mounted with anti-fade reagent and imaged using a confocal laser microscope.

### 2.8. ROS Detection

DCFH-DA is a molecular probe that is intrinsically non-fluorescent and freely permeable to cell membranes. Upon entering cells, it is hydrolyzed by intracellular esterases to yield DCFH, which cannot cross cell membranes. This membrane impermeability facilitates efficient intracellular loading of the probe. Intracellular reactive oxygen species (ROS) then oxidize non-fluorescent DCFH to generate fluorescent DCF. The fluorescence intensity of DCF serves as an indicator for determining intracellular ROS levels.

Intracellular ROS levels were measured using 2′,7′-dichlorodihydrofluorescein diacetate (DCFH-DA, S0033S, Beyotime, Shanghai, China). bEECs were incubated with 10 μM DCFH-DA in serum-free medium for 30 min. Rosup-treated cells served as positive controls. Fluorescence was visualized using a Nikon fluorescence microscope (Tokyo, Japan).

### 2.9. Statistical Analysis

All experiments were repeated three times, and the results are expressed as the mean ± standard error. Data were analyzed and graphed using GraphPad Prism version 8. Non-parametric *t*-tests and one-way ANOVA were performed to assess the significance of differences. Intergroup differences were assessed for statistical significance using Tukey’s multiple comparison test. *p* < 0.05 was considered statistically significant.

## 3. Results

### 3.1. Detection of Inflammatory and Oxidative-Stress-Related Factors in Bovine Uterine Tissues

**Figure 1:**
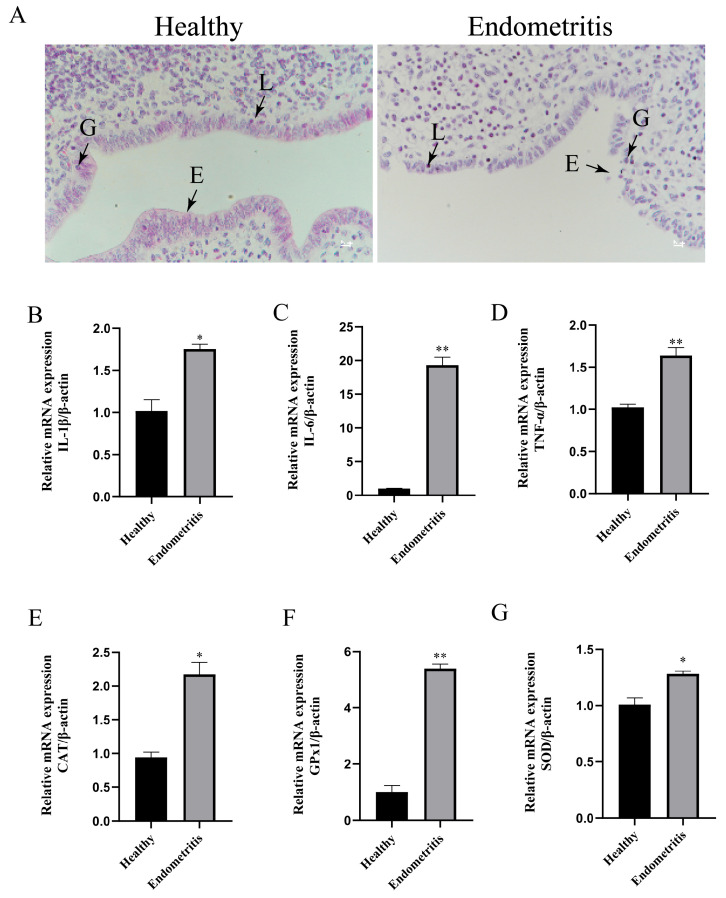
Detection of inflammatory factors and antioxidant-related enzymes in the healthy and endometritis groups of dairy cows. (**A**) Histopathological sections of the healthy group and subclinical group. (**B**–**D**) RT-qPCR analysis of IL-1β, IL-6, and TNF-α mRNA expressions. (**E**–**G**) RT-qPCR analysis of the relative mRNA expression levels of CAT, GPx1, and SOD in bovine uterine tissues. E denotes endometrial epithelial cells, G denotes granulocytes, and L indicates lymphocytes. Scale bar = 100 μm. RT-qPCR data were analyzed using *t*-tests compared to the control group. Significant differences are marked as * *p* < 0.05, ** *p* < 0.01.

Hematoxylin and eosin (HE) staining of the uterine tissues from the healthy group and the endometritis group revealed distinct histological features. In the healthy group, the endometrial epithelial cells exhibited intact morphology with well-aligned arrangements, and minimal infiltration of the lymphocytes and neutrophils was observed in the endometrial epithelium. In contrast, the endometritis group showed severe morphological defects in the endometrial epithelial cells, disordered cellular alignment, and significantly increased infiltration of the lymphocytes and granulocytes in both the endometrial epithelium and stromal regions (Figure 1A). The RT-qPCR analysis of the inflammatory cytokines (IL-1β, IL-6, TNF-α) in the two groups demonstrated that the mRNA expression levels of IL-1β, IL-6, and TNF-α were significantly upregulated in the endometritis group compared to the healthy group (* *p* < 0.05; ** *p* < 0.01) (Figure 1B–D). Furthermore, the RT-qPCR evaluation of the antioxidant-stress-related enzymes (CAT, GPx1, SOD) at the mRNA level indicated that the endometritis group also displayed a marked increase in the mRNA expressions of CAT, GPx1, and SOD compared to the healthy group (Figure 1E–G).

### 3.2. Detection of TGF-β1/Smad3 Signaling Pathway and ECM-Remodeling-Related Factors in Bovine Uterine Tissues

**Figure 2:**
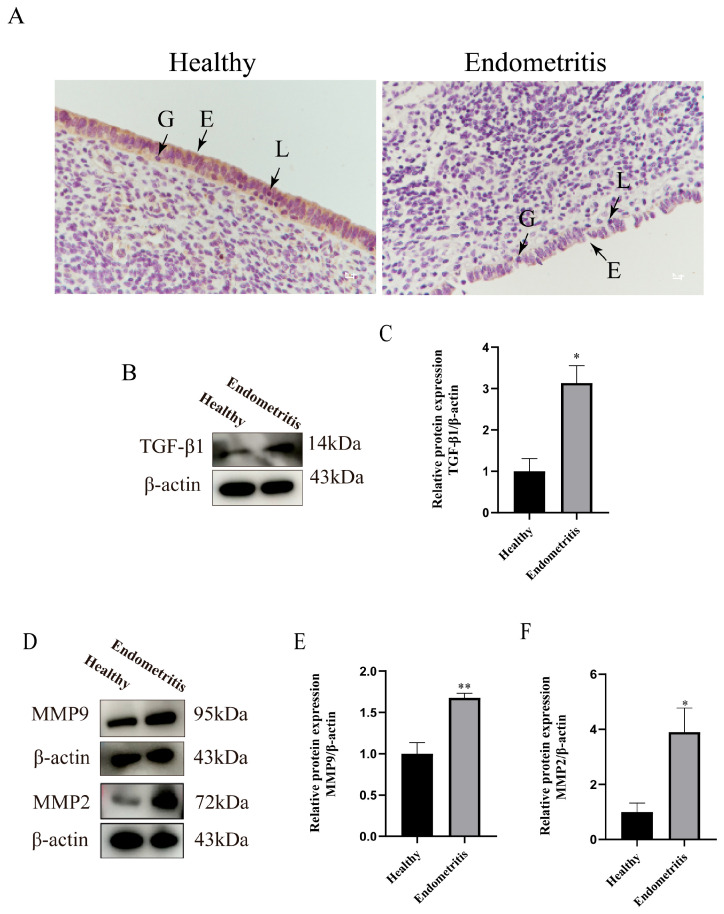
Detection of TGF-β1/Smad3 signaling pathway and ECM-remodeling-related protein expression in the healthy and endometritis groups of dairy cows. (**A**) Immunohistochemical (IHC) analysis of COL-IV protein expression in the endometrial epithelium. (**B**,**C**) Relative expression level of TGF-β1 to β-actin. (**D**–**F**) WB analysis of relative expression level of MMP2 and MMP9 to β-actin. E denotes endometrial epithelial cells, G represents granulocytes, and L indicates lymphocytes. Scale bar = 100 μm. Experiments were repeated three times. Data were analyzed using *t*-tests compared to the control group. Significant differences are marked as * *p* < 0.05, ** *p* < 0.01. Original western blots are presented in File S1.

To investigate the regulatory mechanisms of the TGF-β1/Smad3 signaling pathway and extracellular matrix (ECM) remodeling in bovine endometritis, this study compared the expression differences of related factors between the two groups. COL-IV, a major component of the basement membrane, is synthesized and secreted primarily by endometrial epithelial cells. The basement membrane, located beneath the epithelial layer, provides structural support and connectivity for the epithelial cells. Immunohistochemical staining specific to COL-IV of uterine tissue sections revealed that the protein expression level of COL-IV in the endometrial epithelium of the endometritis group was significantly decreased compared to the control group (Figure 2A). Western blot analysis demonstrated a marked increase in TGF-β1 protein expression in the endometritis group (*p* < 0.05) (Figure 2B,C). Additionally, the expression levels of matrix metalloproteinases MMP2 and MMP9 were significantly upregulated (*p* < 0.05) in the endometritis group (Figure 2D–F).

### 3.3. H_2_O_2_ Activates Inflammation and Oxidative Stress in bEECs

**Figure 3:**
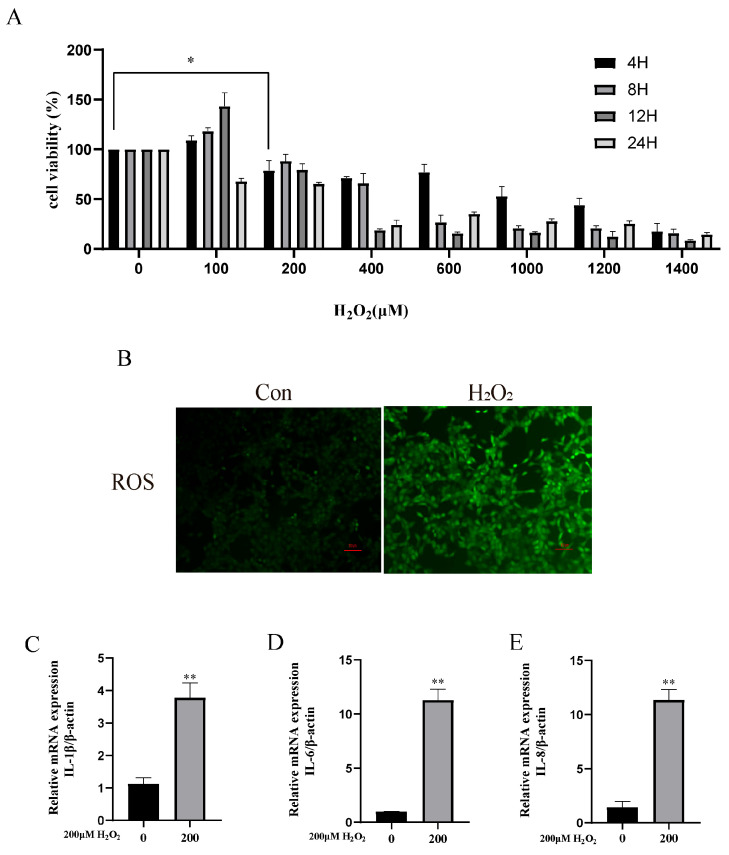
Activation of inflammation and oxidative stress in bEECs by H_2_O_2_. (**A**) CCK-8 assay for cell viability. (**B**) Relative ROS levels. (**C**–**E**) Changes in mRNA expressions of IL-1β, IL-6, and IL-8. Scale bar = 100 μm. Data were analyzed using *t*-tests compared to the control group. Significant differences are marked as * *p* < 0.05 ** *p* < 0.01.

To simulate the in vivo microenvironment, this study utilized CCK-8 to screen H_2_O_2_ treatment concentrations, selecting the condition with 80% cell viability—specifically a 200 μM H_2_O_2_ treatment for 4 h on bEECs—to establish an in vitro inflammatory model, which was subsequently validated (Figure 3A). The results showed that the H_2_O_2_-treated group exhibited a significant increase in reactive oxygen species (ROS) compared to the control group (Figure 3B). The RT-qPCR analysis revealed that the mRNA expression levels of IL-1β, IL-6, and IL-8 were markedly elevated in the treated group compared to the control group (** *p* < 0.01) (Figure 3C–E).

### 3.4. H_2_O_2_ Activates the TGF-β1/Smad3 Pathway to Enhance ECM Remodeling

**Figure 4:**
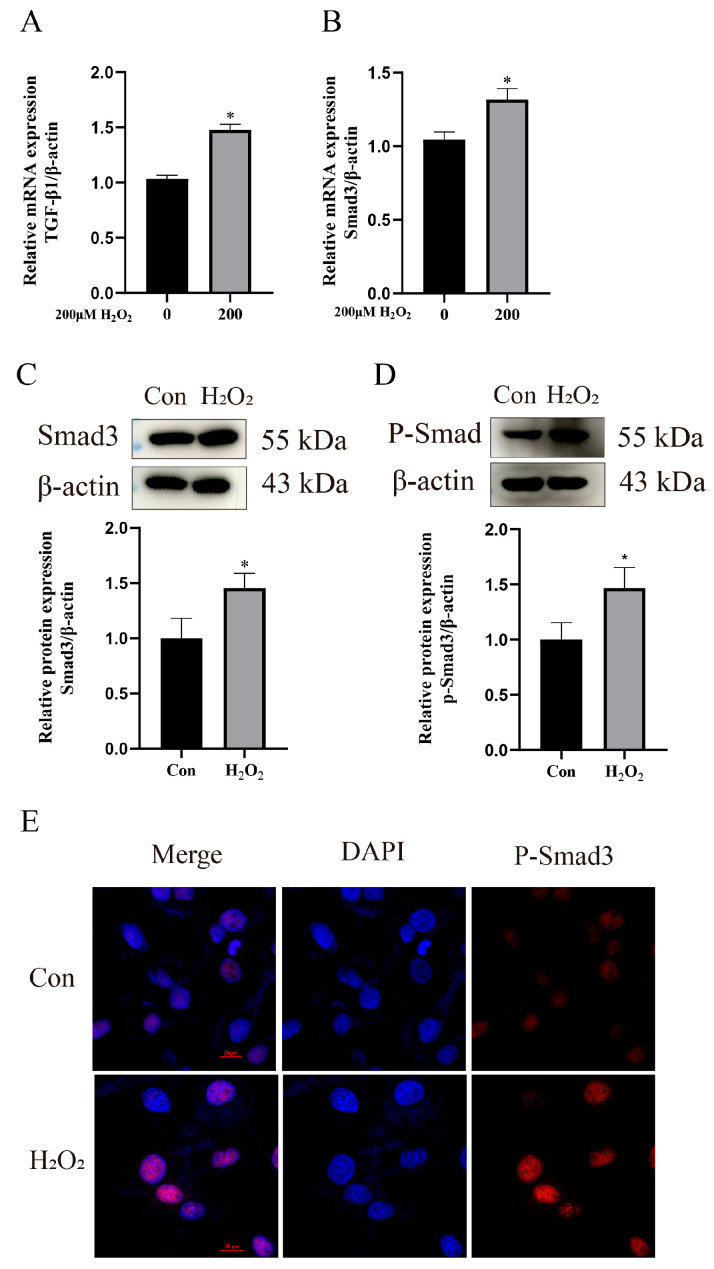
Activation of TGFβ1/Smad3 pathway by H_2_O_2_. (**A**) RT-qPCR analysis of TGFβ1 mRNA expression. (**B**,**C**) RT-qPCR and Western blot analysis of Smad3 expression. (**D**) Relative expression level of P-Smad3 to β-actin (**E**) Immunofluorescence detection of nuclear P-Smad3 protein localization. Scale bar = 20 μm. Data were analyzed using *t*-tests compared to control group. Significant differences are marked as * *p* < 0.05. Original western blots are presented in File S1.

To validate whether this in vitro model activated the TGFβ1/Smad3 pathway, the expression of related factors was examined. The RT-qPCR results demonstrated that TGFβ1 mRNA expression was significantly upregulated in the H_2_O_2_-treated group compared to the control group (*p* < 0.05) (Figure 4A). Both the Smad3 mRNA and protein expression levels were significantly increased (*p* < 0.05) (Figure 4B,C). Furthermore, Western blot analysis revealed upregulated phosphorylated Smad3 (P-Smad3) expression (*p* < 0.05), and immunofluorescence staining showed enhanced nuclear localization of P-Smad3 (Figure 4D,E).

**Figure 5:**
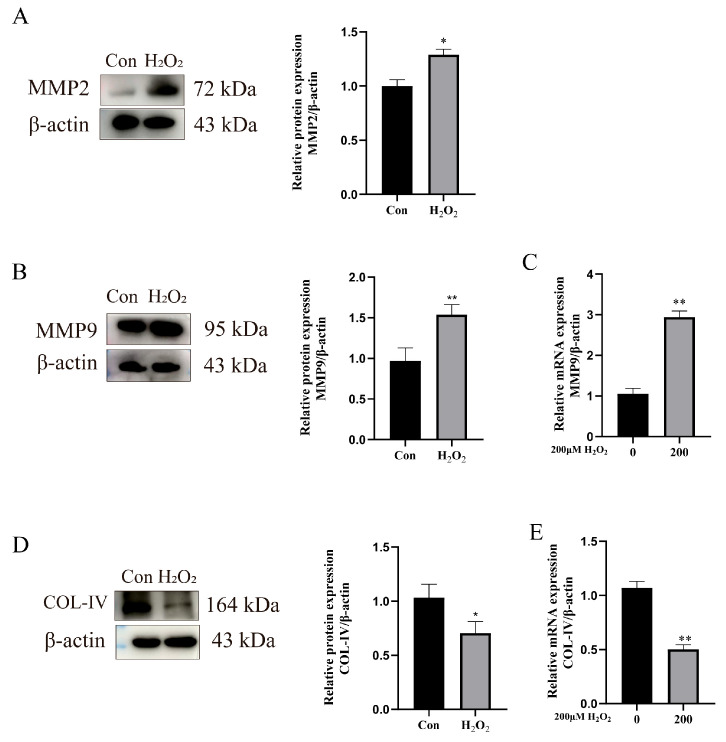
Activation of ECM remodeling by H_2_O_2_. (**A**) Relative expression level of MMP2 to β-actin. (**B**,**C**) RT-qPCR and Western blot analysis of MMP9 expression. (**D**,**E**) RT-qPCR and Western blot analysis of COL-IV expression. Data were analyzed using *t*-tests compared to control group. Significant differences are marked as * *p* < 0.05, ** *p* < 0.01. Original western blots are presented in File S1.

To investigate whether the in vitro inflammatory model activates extracellular matrix (ECM) remodeling, this study examined the expression of ECM-remodeling-related factors. The Western blot analysis of MMP2 expression showed a significant increase in the MMP2 mRNA levels (*p* < 0.05) (Figure 5A). Both the RT-qPCR and Western blot analyses revealed the upregulated mRNA and protein expression of MMP9 (*p* * < 0.01) (Figure 5B,C). In contrast, the mRNA and protein expression levels of COL-IV were significantly decreased (*p* * < 0.01) (Figure 5D,E).

### 3.5. Impact of TGF-β1/Smad3 Pathway Expression on ECM Remodeling-Related Proteins

**Figure 6:**
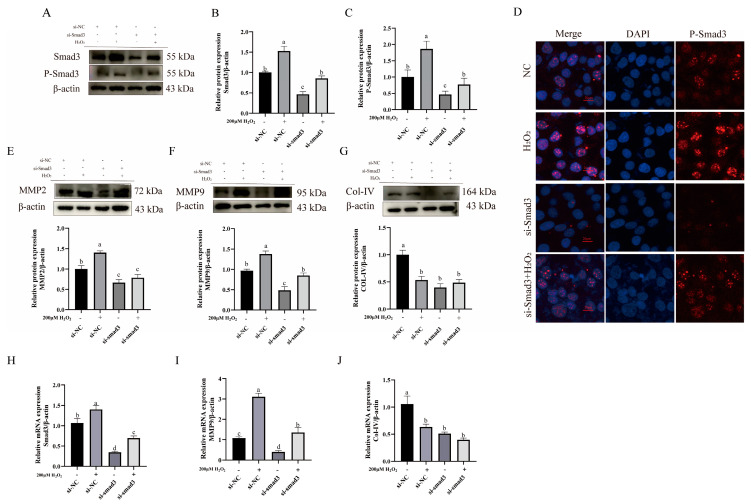
Effects of TGFβ1/SMAD3 pathway expression on ECM-remodeling-related protein levels. (**A**–**C**) Western blot (WB) analysis of relative expression of Smad3 and phosphorylated Smad3 (P-Smad3). (**D**) Immunohistochemical (IHC) staining showing nuclear localization of P-Smad3. (**E**–**G**) WB analysis of relative expressions of MMP2, MMP9, and COL-IV. (**H**–**J**) RT-qPCR analysis of relative mRNA expressions of Smad3, MMP9 (**H**), and COL-IV. Scale bar = 20 μm. Data were analyzed using one-way ANOVA compared to control group. Different lowercase letters indicate statistically significant differences (*p* < 0.05). Original western blots are presented in File S1.

To elucidate the role of the TGF-β1/Smad3 signaling pathway in extracellular matrix (ECM) remodeling, this study interfered with Smad3 expression and examined the expression of the downstream ECM-remodeling-related proteins. The results showed that compared to the control and H_2_O_2_-treated groups, the si-Smad3-treated group exhibited significant downregulation of Smad3 protein expression (*p* < 0.05) (Figure 6A,B). Concurrently, the Western blot (WB) analysis revealed a marked reduction in phosphorylated Smad3 (P-Smad3) levels (*p* < 0.05) (Figure 6A,C). Immunohistochemical staining further demonstrated decreased nuclear expression of P-Smad3 (*p* < 0.05) (Figure 6D). However, the expression levels of Smad3 and P-Smad3 in the combination treatment group showed no significant changes compared to the control group.

Following the downregulation of Smad3 and P-Smad3, the MMP9 protein levels significantly reduced (*p* < 0.05) (Figure 6E), MMP2 protein expression was notably downregulated (*p* < 0.05) (Figure 6F), and COL-IV protein expression further decreased (*p* < 0.05) (Figure 6G). At this stage, MMP2 expression in the combination treatment group was significantly downregulated compared to the control group (*p* < 0.05), while showing no significant difference from the Smad3 knockdown group (Figure 6E). The combination treatment group exhibited no significant change in MMP9 expression relative to the controls but demonstrated significant upregulation compared to the Smad3 knockdown group (*p* < 0.05) (Figure 6F). The COL-IV expression in the combination treatment group was significantly downregulated versus the control group (*p* < 0.05) (Figure 6G), with no significant alteration observed relative to the Smad3 knockdown group.

Additionally, bEECs were pretreated with sb431542, a TGF-β1/Smad3 pathway inhibitor. The RT-qPCR analysis confirmed that the sb431542-treated group successfully downregulated Smad3 mRNA expression compared to the control and H_2_O_2_-treated groups (*p* < 0.05) (Figure 6H). Simultaneously, the MMP9 mRNA expression was significantly suppressed (*p* < 0.05) (Figure 6I), while the COL-IV expression was markedly reduced compared to the control group (*p* < 0.05) (Figure 6J). At this stage, the MMP2 and MMP9 expressions in the combination treatment group demonstrated a partially rescued increase compared to the Smad3 knockdown group (*p* < 0.05) (Figure 6I), whereas the COL-IV expression showed no significant difference relative to the Smad3 knockdown group.

These observations indicate that upon blockade of the TGF-β1/Smad3 pathway, the expression of COL-IV-degrading enzymes (MMP2 and MMP9) is downregulated, while COL-IV shows no compensatory upregulation. This demonstrates the pathway’s dual regulatory effects on both targets. Furthermore, H_2_O_2_ partially modulates this regulatory process.

## 4. Discussion

Endometritis is a common postpartum disease in dairy cows, significantly impairing reproductive performance and causing substantial economic losses. The pathological progression of endometritis varies among individuals: some affected cows recover spontaneously, while others develop incomplete uterine involution, though the underlying mechanisms remain unclear. This study aims to elucidate how the TGF-β1/Smad3 signaling pathway regulates ECM remodeling in H_2_O_2_-mediated bovine endometritis.

In ruminants, ECM remodeling plays critical roles in reproductive physiology and mammary gland development/involution [28]. Studies demonstrate that during bovine uterine involution, TGF-β regulates ECM remodeling by modulating the MMP/TIMP balance, with postpartum dysregulation of the MMP9/TIMP1 ratio causing delayed conception [29]. In dairy cow mastitis, TGF-β1 significantly promotes mammary fibrosis, accompanied by substantial ROS generation [30]. Notably, dynamic alterations in COL-IV occur during bovine embryo implantation, indicating its essential functional contribution to this process [31]. Infertility caused by endometritis primarily arises from incomplete uterine involution in affected cows, though the precise mechanisms remain poorly understood. Current research demonstrates significant changes in collagen expression during uterine involution, with matrix metalloproteinases (MMP2 and MMP9) playing pivotal roles [32].

Oxidative stress and endometritis exhibit reciprocal causation. A tightly interdependent bidirectional relationship exists between inflammation and oxidative stress, wherein these processes amplify each other through complex molecular networks to form a vicious cycle that collectively drives the pathogenesis of multiple diseases. Primarily, oxidative stress induces inflammation: ROS activates multiple signaling pathways to provoke inflammatory responses. For instance, ROS-mediated oxidation inhibits IκB, leading to NF-κB nuclear translocation and the subsequent transcription of the TNF-α and IL-6 genes. Additionally, ROS directly oxidizes the NLRP3 protein to promote inflammasome assembly, thereby triggering IL-1β/IL-18 release [33].Conversely, inflammation activates oxidative stress: Membrane-bound NADPH oxidase (NOX) in activated immune cells generates substantial superoxide anions (O_2_•^−^), while inflammatory cytokines stimulate mitochondrial electron transport chains to increase ROS production [34]. Postpartum oxidative stress levels rise in cows, coinciding with immunosuppression and the transition of the reproductive tract from a closed to an open state [35]. Under these conditions, pathogens can invade the uterus through the birth canal, predisposing cows to endometritis. Studies indicate that inflammatory tissues often exhibit abnormal antioxidant enzyme expression. For instance, in acute lung injury models, inflammatory cells release ROS that activate the Nrf2 pathway, inducing SOD and CAT expression [36]. In ulcerative colitis, the expressions of CAT and GPx are significantly upregulated [37]. In this study, the histopathological examination of bovine uterine tissues and RT-qPCR analysis of inflammatory cytokines (IL-1β, IL-6, TNF-α) revealed that the endometritis group exhibited significantly upregulated expressions of antioxidant stress factors (GPx1, SOD, CAT) (*p* < 0.05), consistent with prior reports.

Under physiological conditions, inflammation and oxidative stress collaboratively regulate ECM remodeling. This coordination operates primarily through the modulation of collagen expression and degradation by MMPs. For example, MMP9 upregulation has been documented in rheumatoid arthritis [38], while increased MMP2/9 expression facilitates resolution of pulmonary inflammation and fibrosis in patients with COVID-19 [39]. Conversely, inflammatory cytokines and ROS directly modulate COL-IV expression. As evidenced in diabetic nephropathy, the pro-inflammatory cytokine TNF-α suppresses COL4A1 synthesis through the IRE1α/XBP1 endoplasmic reticulum stress pathway [40]. Additionally, the TGFβ1/Smad3 pathway is broadly involved in MMP2/9 regulation. In breast cancer, lactate modulates cancer cell invasion via the TGFβ1/Smad3/MMP2/9 signaling axis [41]. Simultaneously, latent TGF-β serves as the primary substrate for MMP2 and MMP9. These proteases specifically cleave the linker regions within LAP (latency-associated peptide) or LAP-LTBP (latent TGF-β-binding protein) complexes. This proteolytic cleavage disrupts the structural integrity of the latent complex, releasing bioactive mature TGF-β molecules that in turn amplify TGF-β expression [42]. This study found upregulated expressions of TGFβ1, Smad3, and MMP2/9 during bovine endometritis.

Type IV collagen (COL-IV), a major basement membrane component synthesized intracellularly, directly contributes to extracellular matrix formation [43]. MMP2 and MMP9 degrade COL-IV by cleaving its triple-helical structure [44]. Intriguingly, COL-IV undergoes divergent fates—either degradation or deposition—across distinct inflammatory contexts. During acute inflammation, the ECM predominantly undergoes net degradation. For instance, in hemophilic synovitis, MMP2/9 upregulation reduces COL-IV expression in the vascular basement membranes, increasing vascular permeability [45]. Similarly, during atherosclerosis, macrophage-derived ROS upregulate MMP2/9 to degrade COL-IV, promoting plaque rupture [46]. In contrast, chronic inflammatory processes often show opposing COL-IV trends. Chronic liver injury induces ECM deposition, including COL-IV, leading to fibrosis [47], while COL-IV overexpression in chronic kidney disease drives renal interstitial fibrosis [48]. In this study, COL-IV expression was downregulated in the endometritis tissues, aligning with existing findings.

H_2_O_2_ is widely used to establish in vitro cellular oxidative stress models. Previous studies in our laboratory demonstrated that treating bEECs with 50, 100, or 200 μM H_2_O_2_ effectively induces reactive oxygen species (ROS) and inflammatory factor expression [27]. In this study, an in vitro inflammatory model was constructed by treating bEECs with 200 μM H_2_O_2_, resulting in the upregulated expression of inflammatory cytokines IL-1β, IL-6, and IL-8. Studies indicate that H_2_O_2_ activates the TGFβ1/Smad3 signaling pathway. For instance, H_2_O_2_ enhances TGFβ1 gene expression by activating AP-1 and NF-κB, as shown in lung fibroblasts, where H_2_O_2_ stimulation increases TGFβ1 secretion 2–3-fold [49]. Additionally, H_2_O_2_ amplifies TGFβRI-mediated Smad3 phosphorylation by activating TAK1 (TGFβ-activated kinase 1) or inhibiting protein phosphatases such as PP2A [50]. Our findings reveal that H_2_O_2_ significantly elevates TGFβ1 expression (*p* < 0.05) and upregulates its downstream proteins Smad3 and phosphorylated Smad3 (P-Smad3) (*p* < 0.05) in bEECs, confirming the successful activation of the TGFβ1/Smad3 pathway.

Recent studies demonstrate that H_2_O_2_ treatment activates ECM remodeling. H_2_O_2_ enhances MMP2 and MMP9 gene expression via AP-1 and NF-κB activation [51]. Furthermore, H_2_O_2_ directly regulates MMP2 by oxidizing the cysteine switch domain of its proenzyme, promoting autolytic activation, with MMP2 activity doubling under H_2_O_2_ stimulation [52]. In this study, H_2_O_2_-treated bEECs exhibited significantly increased expression of ECM-remodeling-related proteins MMP2 and MMP9 (*p* < 0.05) and decreased COL-IV expression (*p* < 0.01), aligning with prior tissue-level observations. These results indicate the concurrent activation of the TGFβ1/Smad3 pathway and ECM remodeling in oxidative-stress-mediated endometritis.

The TGFβ1/Smad3 pathway plays a critical role in ECM remodeling. Studies suggest it regulates COL-IV through two mechanisms: (1) The first mechanism is MMP2/MMP9-mediated degradation. Upon TGFβ1 activation, phosphorylated Smad3 forms a complex with Smad4, binding directly to Smad-binding elements (SBEs) in the promoters of MMP2 and MMP9 to enhance their transcription. For example, in hepatocellular carcinoma cells, TGFβ1 upregulates MMP2 via Smad3, boosting invasiveness [53]. Similarly, Smad3 knockout in breast cancer models blocks TGFβ1-induced MMP9 expression, inhibiting metastasis [54]. (2) The second mechanism is the direct regulation of COL-IV synthesis. In diabetic nephropathy, where COL-IV is a major deposited collagen, Smad3 binds to promoter regions of COL4A1A2, COL4A3A4, and COL4A5, a process inhibited by C-peptide [55].

Our experimental results demonstrate that the H_2_O_2_-induced activation of the TGFβ1/Smad3 pathway upregulates MMP2/MMP9 expression and degrades COL-IV (*p* < 0.01). Conversely, inhibiting the TGFβ1/Smad3 pathway reduces Smad3 and P-Smad3 levels (*p* < 0.05), suppresses MMP2/MMP9 promoter activity (*p* < 0.05), and further diminishes COL-IV transcription due to disrupted Smad3 binding (*p* < 0.05). This dual regulatory mechanism highlights how the TGFβ1/Smad3 pathway governs COL-IV dynamics in H_2_O_2_-induced bEEC inflammation.

Based on the above findings, we speculate that the TGF-β1/Smad3 pathway may exert a dual regulatory effect on ECM remodeling: suppressing this pathway can reduce the expression of COL-IV-degrading enzymes (MMP2/9) while simultaneously inhibiting COL-IV synthesis. Since this study only included Holstein cattle (n = 6), the small sample size and lack of breed diversity may limit the generalizability of the results. Future research will expand sample diversity to validate the broader applicability of these conclusions. Additionally, while the in vitro H_2_O_2_ stimulation model allows variable control, it cannot fully replicate the complex oxidative stress microenvironment in vivo. Future work will return to animal models for further validation of the pathway mechanisms. Despite these limitations, this discovery provides a theoretical foundation for elucidating the molecular mechanisms through which endometritis causes delayed uterine involution.

Based on these findings, the ratio of MMP9 to COL-IV in uterine lavage fluid could serve as a diagnostic indicator to determine the inflammatory phase during clinical assessment. Accordingly, this ratio may guide decisions on whether antioxidant intervention is warranted.

## 5. Conclusions

Our findings demonstrate that oxidative-stress-mediated bovine endometritis activates extracellular matrix (ECM) remodeling. Furthermore, we speculate that oxidative stress may influence the dynamic equilibrium between MMPs and COL-IV through the TGF-β1/Smad3 pathway, thereby exerting bidirectional regulation on ECM remodeling.

## Figures and Tables

**Table 1:** Reaction system for RT-qPCR.

Reagent	Volume (μL)
ChamQ SYBR qPCR Green Master Mix	10.0
Primer (F + R) (10 μM)	1.0
cDNA	4.0
RNase Free dH2O	5.0
Total	20.0

**Table 2:** The primer sequences for RT-qPCR.

Gene Name	ID	Sequence (5′-3′)	Size (bp)
*TNF-α*	NM_173966	F: GCTCTTACCGGAACACTTCG R: GGACACCTTGACCTCCTGAA	238
*IL-1β*	NM_174093	F: AACCGAGAAGTGGTGTTCTGC R: TTGGGGTAGACTTTGGGGTCT	107
*IL-6*	NM_173923.2	F: CTACCTCCAGAACGAGTATG R: CAGCAGGTCAGTGTTTGTGG	136
*IL-8*	NM_173925.2	F: CATTCCACACCTTTCCACCC R: AGGCAGACCTCGTTTCCATT	116
*CAT*	NM_001035386.2	F: AGAGGAAACGCCTGTGTGAG R: ATGCGGGAGCCATATTCAGG	115
*SOD*	NM_174615.2	F: CTCTACTTGGTTGGGGCGTC R: TCGAAGTGGATGGTGCCTTG	122
*GPx1*	NM_174076.3	F: AACGTAGCATCGCTCTGAGG R: GATGCCCAAACTGGTTGCAG	121
*TGF-β1*	NM_001007816.1	F: CCGGAGGACCGAGACCTG R: CGTCAGATACTCCGAGGTGC	97
*Smad3*	XM_005202615.5	F: AGCATAGCACGGAAGCAGG R: GGTCTTATCCAAAGCGTCTGC	87
*MMP9*	NM_174744.2	F: CCATCGCGGAGATTAGGAAC R: CAGGCCACTTGCTCTTGACA	117
*COL-Ⅳ*	XM_025000171.2	F: CGTGCCACTACTACGCGAAC R: CCTTGAGTGTGTCGGCAGAG	93
*ACTB* (*β-Actin*)	NM_173979.3	F: GGCACCCAGCACAATGAAGA R: GCCAATCCACACGGAGTACTT	67

**Table 3:** The primer sequences of Si-RNA.

Gene Name	Sequence (5′-3′)
*Si-NC*	F: UUCUCCGAACGUGUCACGUTT R: ACGUGACACGUUCGGAGAATT
*Si-Smad3*	F: GAGUUCGCCUUCAACAUGATT R: UCAUGUUGAAGGCGAACUCTT

## Data Availability

Data are available upon request due to privacy/ethical restrictions.

## Supplementary Materials

The following supporting information can be downloaded at: https://www.mdpi.com/article/10.3390/ani15131847/s1, File S1: Original western blots.
