# Supplementary figures and images for: Oxidative Stress Mediates the Dual Regulatory Effects of Bovine Uterine ECM Remodeling Through the TGF-β1/Smad3 Pathway: Molecular Mechanisms of MMPs and COL-IV Imbalances
Source: Animals (Basel). 2025 Jun 23;15(13):1847. doi: 10.3390/ani15131847 (PMC12248901; doi:10.3390/ani15131847)

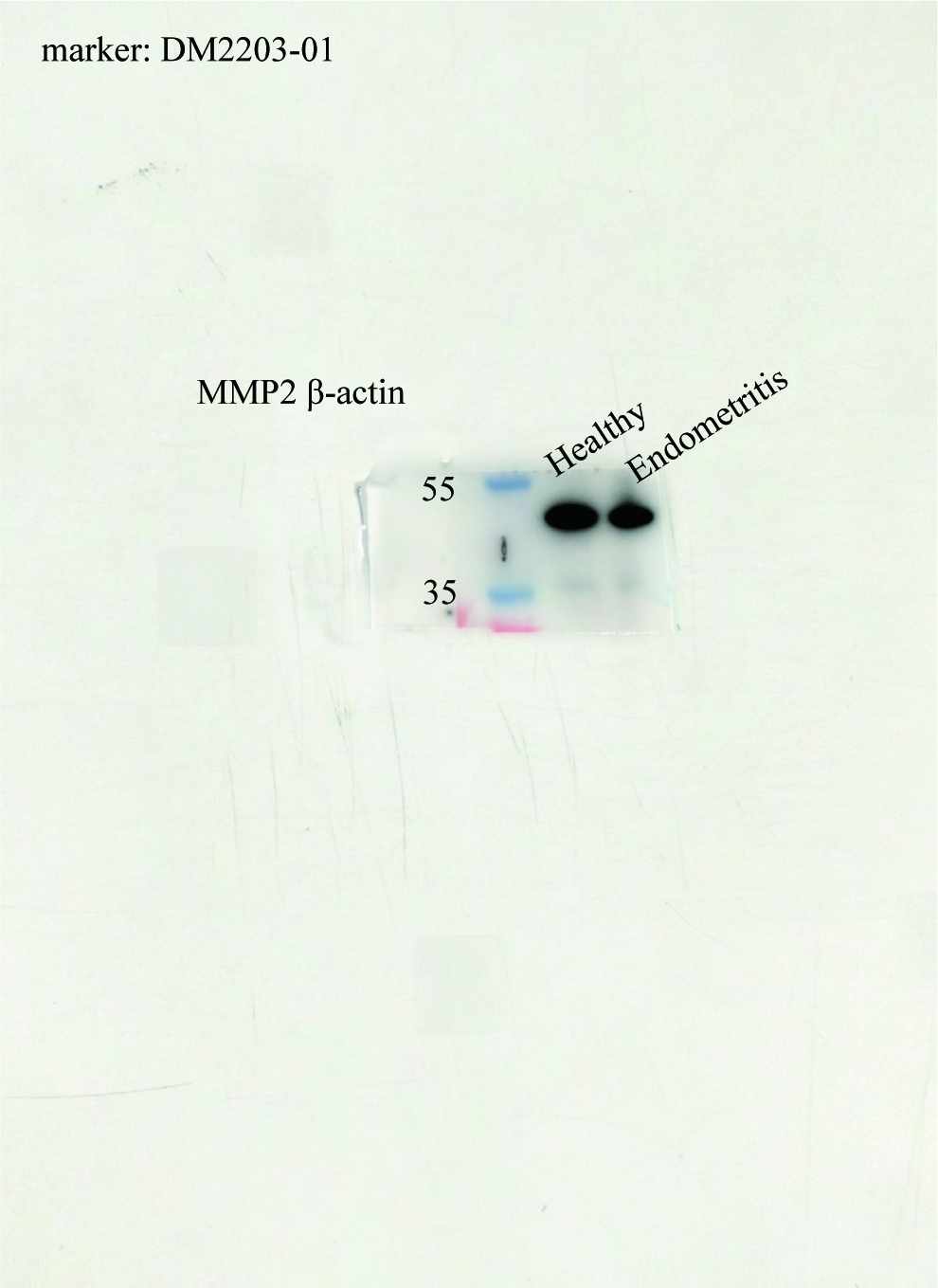

Supplement: Supplementary file 1 [file animals-15-01847-s001.zip › FIG. 2/MMP2 actin.tif]

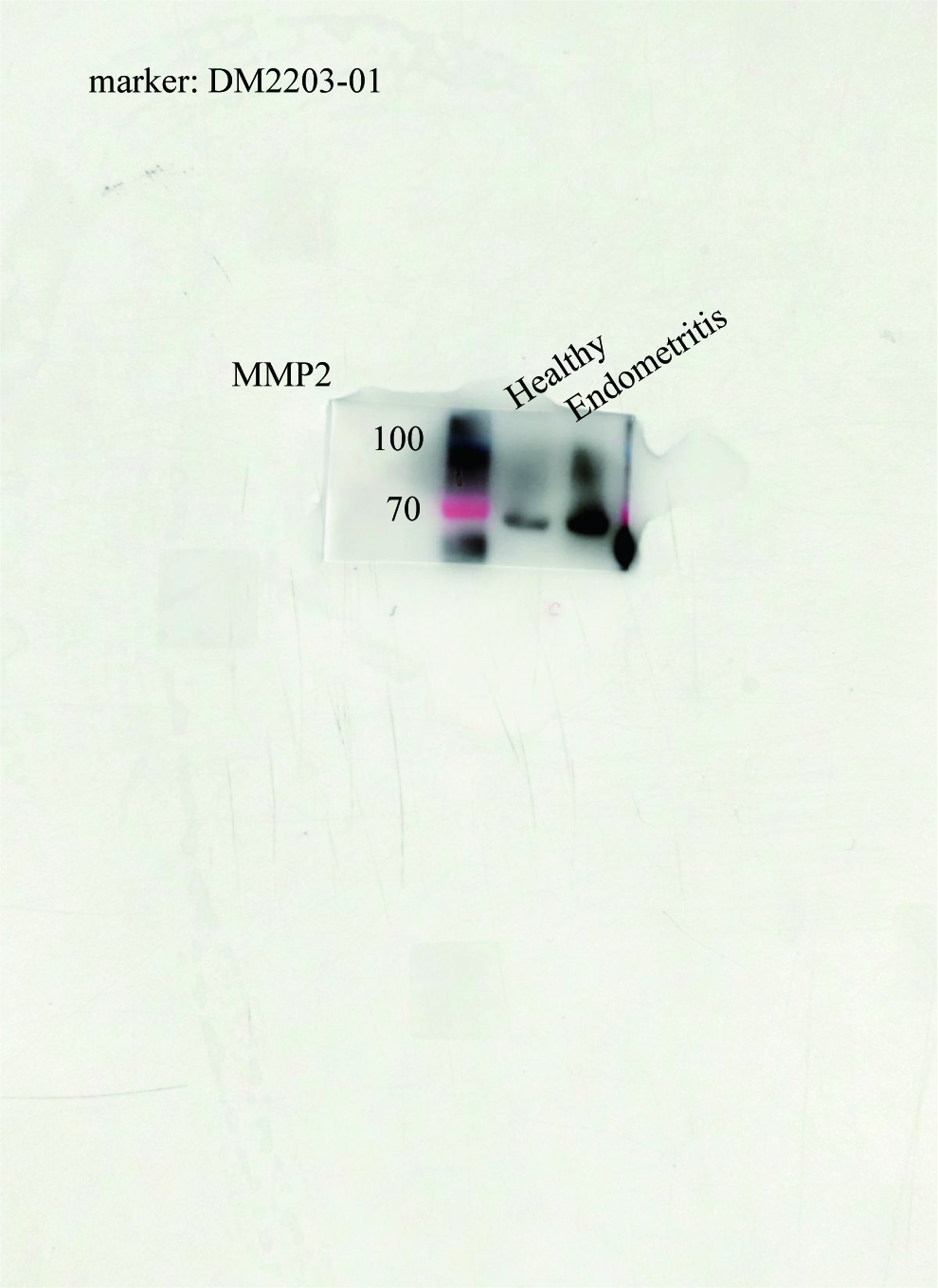

Supplement: Supplementary file 1 [file animals-15-01847-s001.zip › FIG. 2/MMP2.tif]

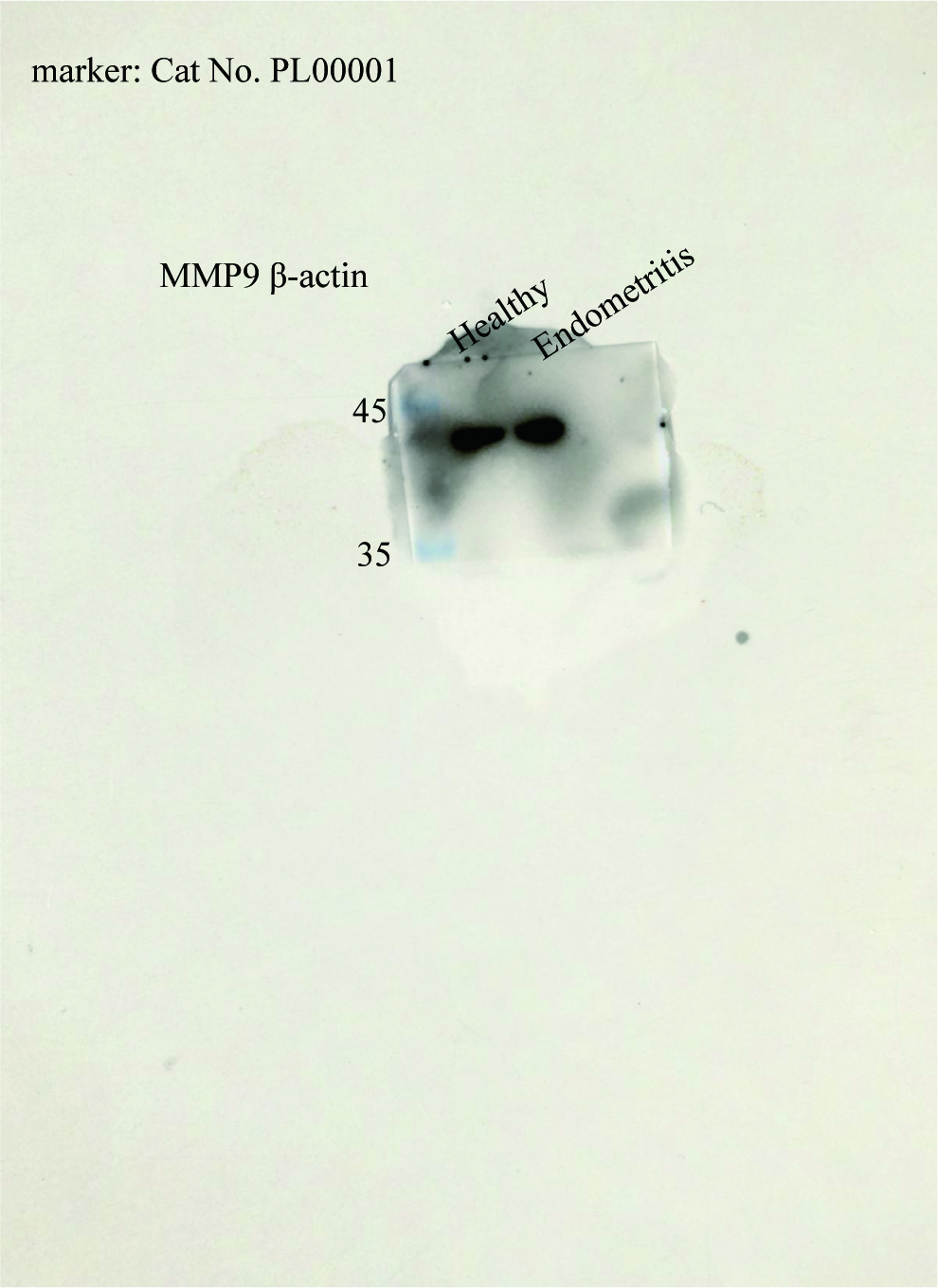

Supplement: Supplementary file 1 [file animals-15-01847-s001.zip › FIG. 2/MMP9 actin.tif]

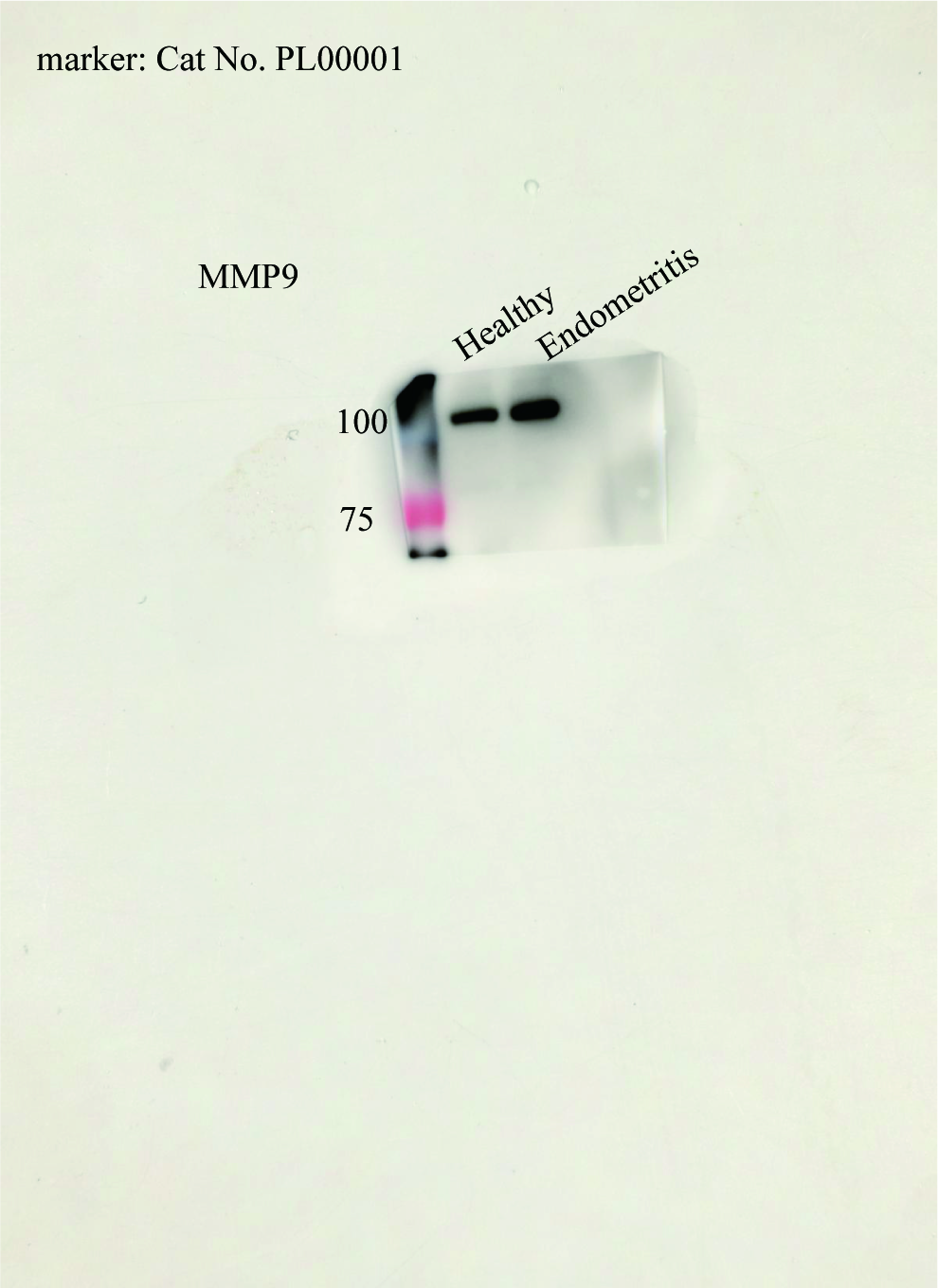

Supplement: Supplementary file 1 [file animals-15-01847-s001.zip › FIG. 2/MMP9.tif]

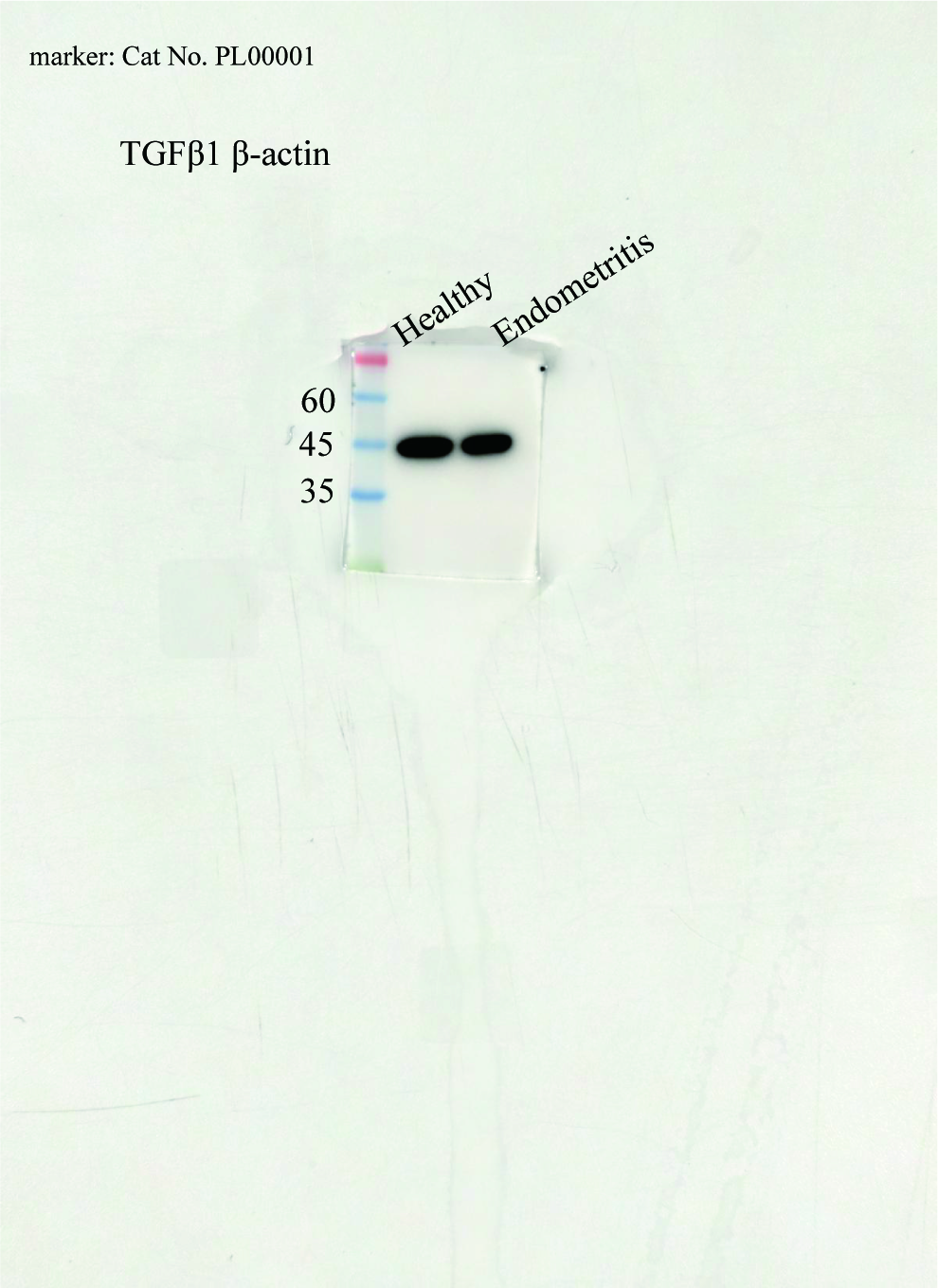

Supplement: Supplementary file 1 [file animals-15-01847-s001.zip › FIG. 2/TGFβ1 actin.tif]

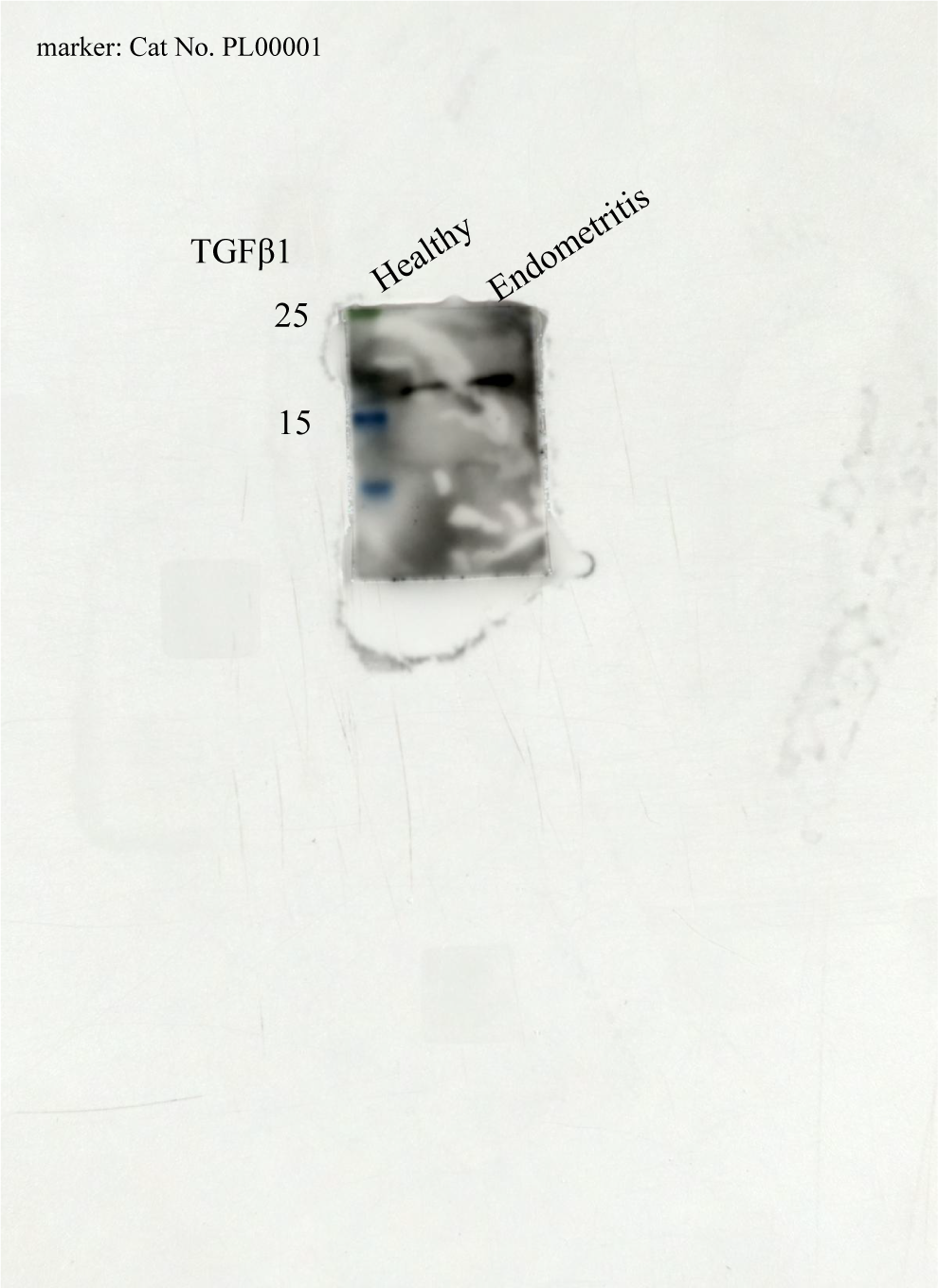

Supplement: Supplementary file 1 [file animals-15-01847-s001.zip › FIG. 2/TGFβ1.tif]

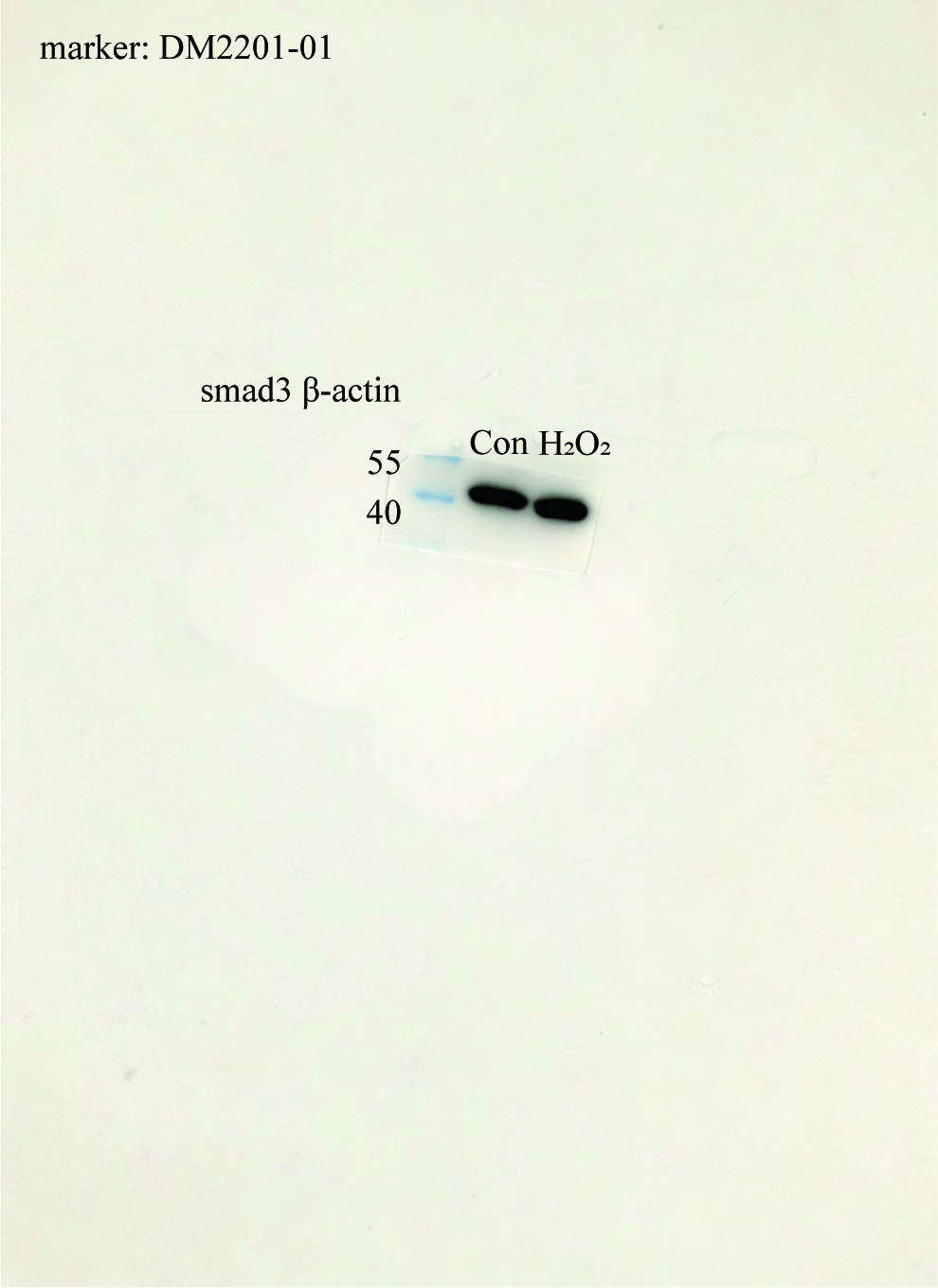

Supplement: Supplementary file 1 [file animals-15-01847-s001.zip › FIG. 4/smad 3 actin.tif]

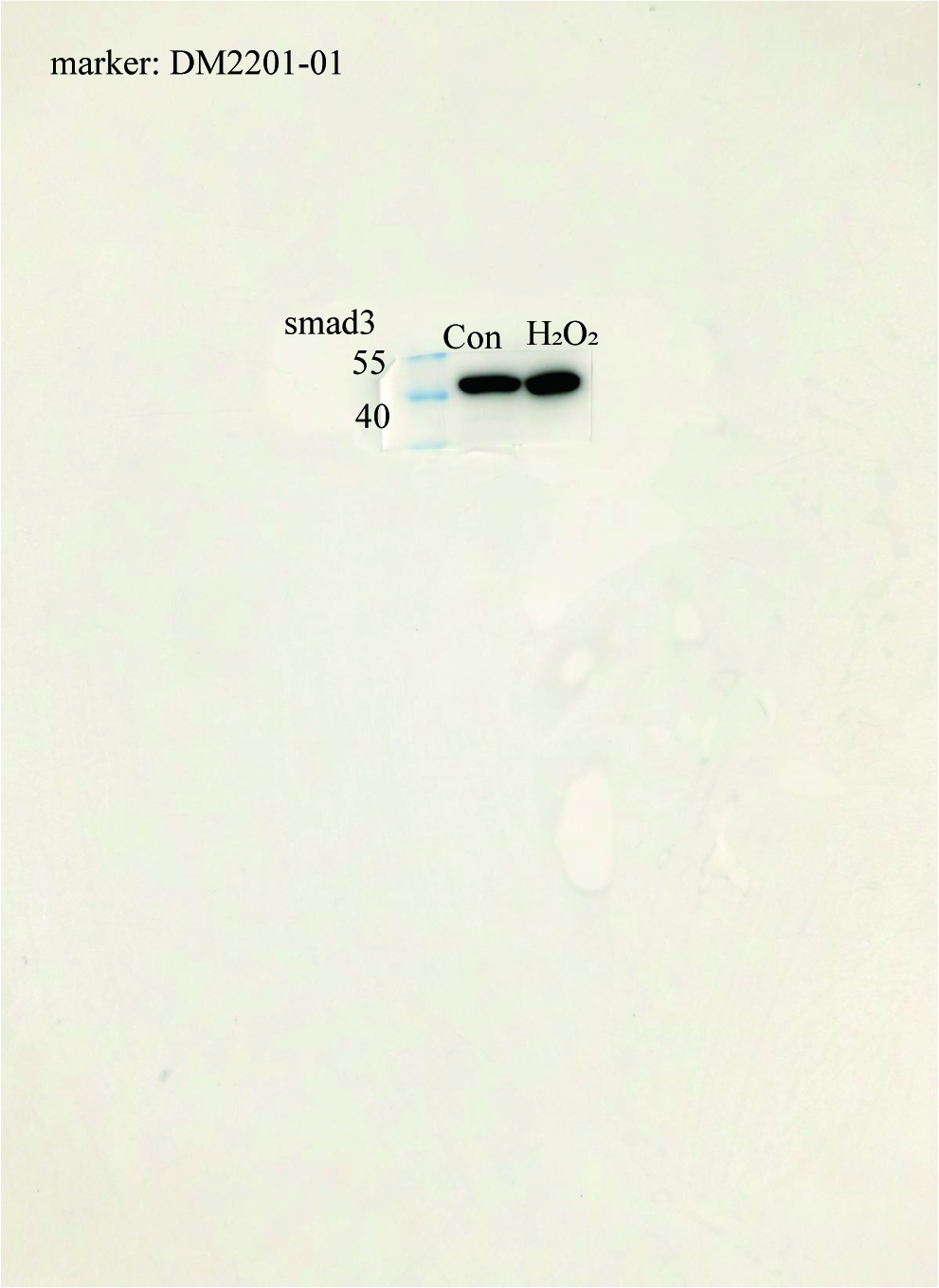

Supplement: Supplementary file 1 [file animals-15-01847-s001.zip › FIG. 4/smad 3.tif]

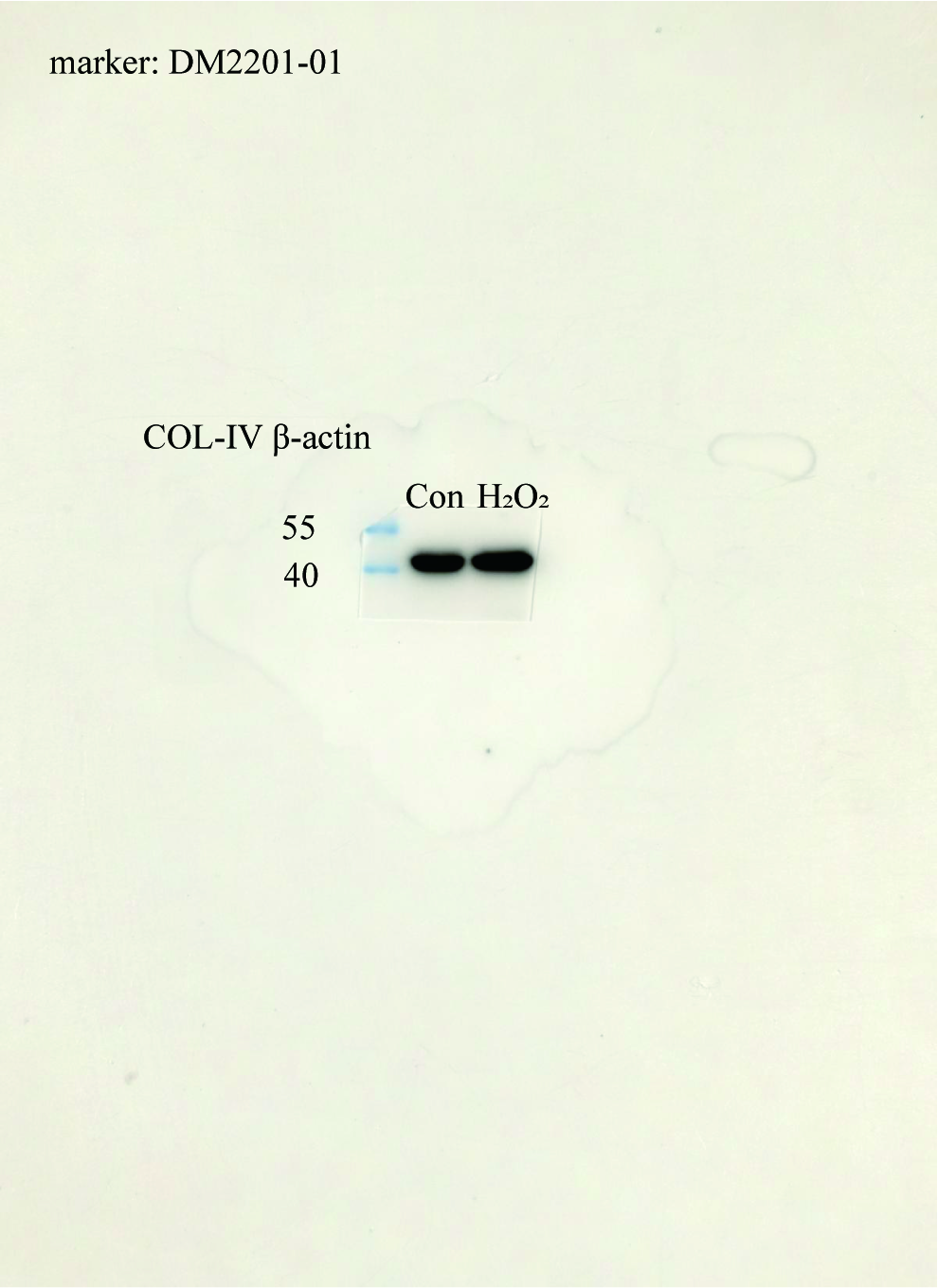

Supplement: Supplementary file 1 [file animals-15-01847-s001.zip › FIG. 5/COL-IV actin.tif]

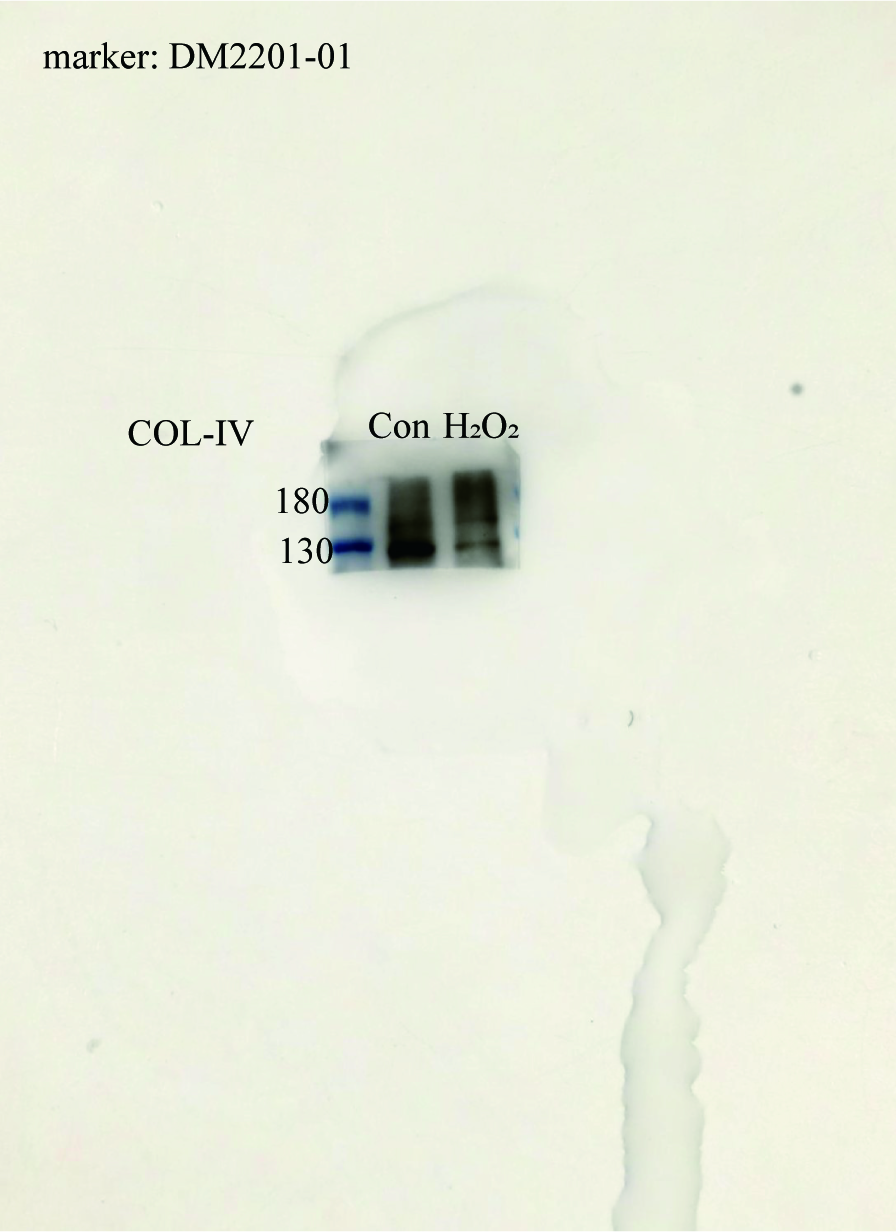

Supplement: Supplementary file 1 [file animals-15-01847-s001.zip › FIG. 5/COL-IV.tif]

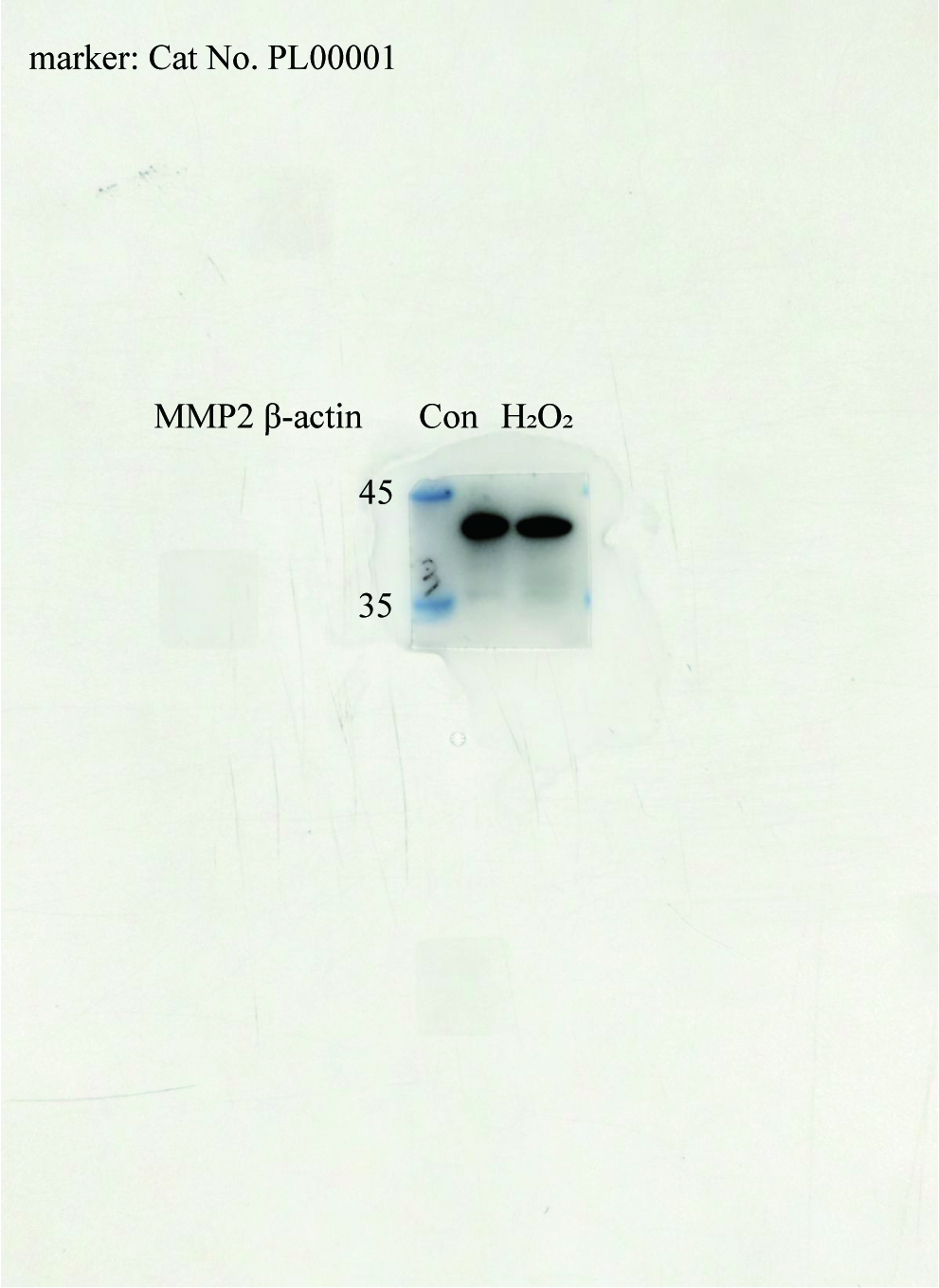

Supplement: Supplementary file 1 [file animals-15-01847-s001.zip › FIG. 5/MMP2 actin.tif]

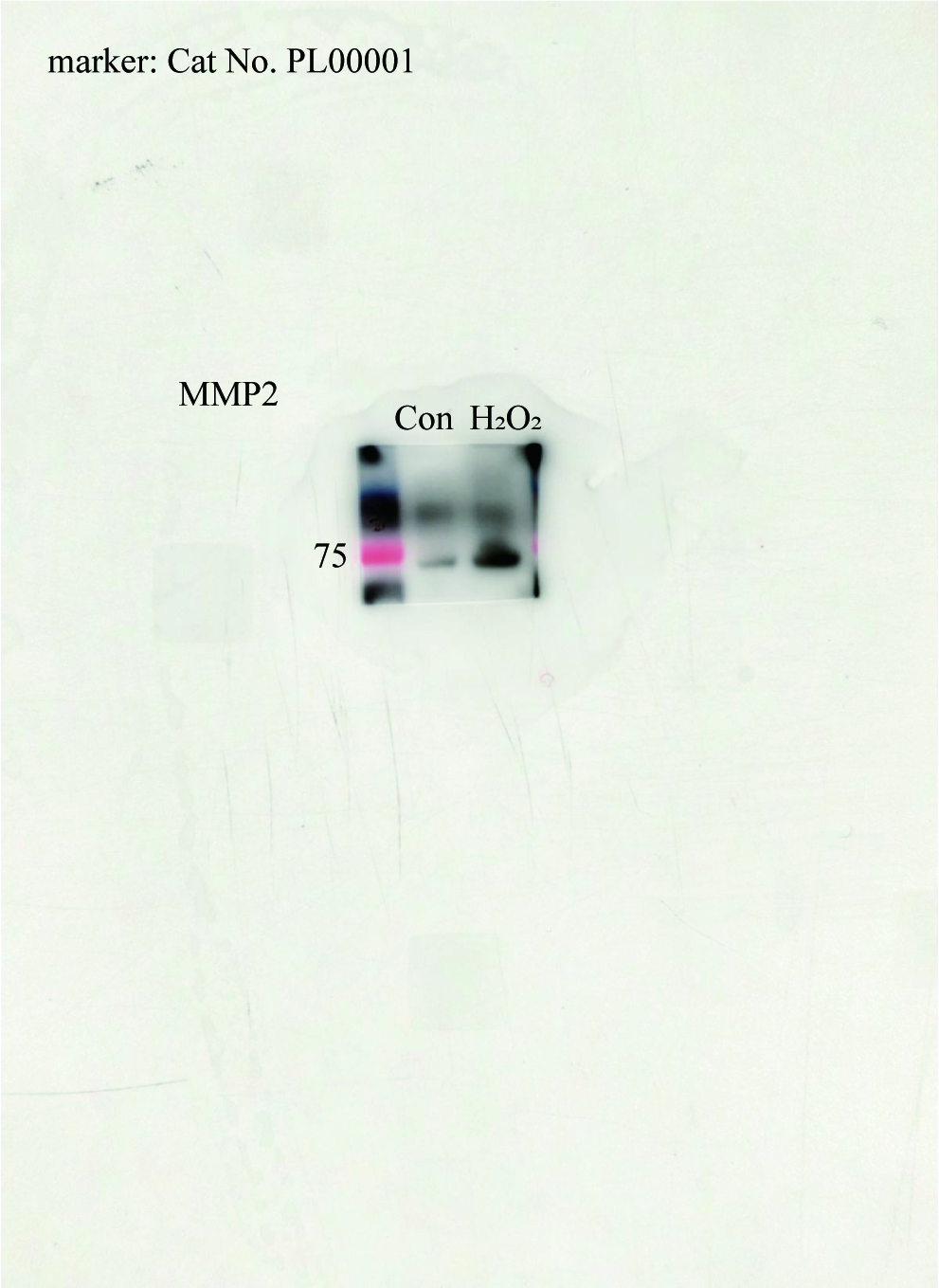

Supplement: Supplementary file 1 [file animals-15-01847-s001.zip › FIG. 5/MMP2.tif]

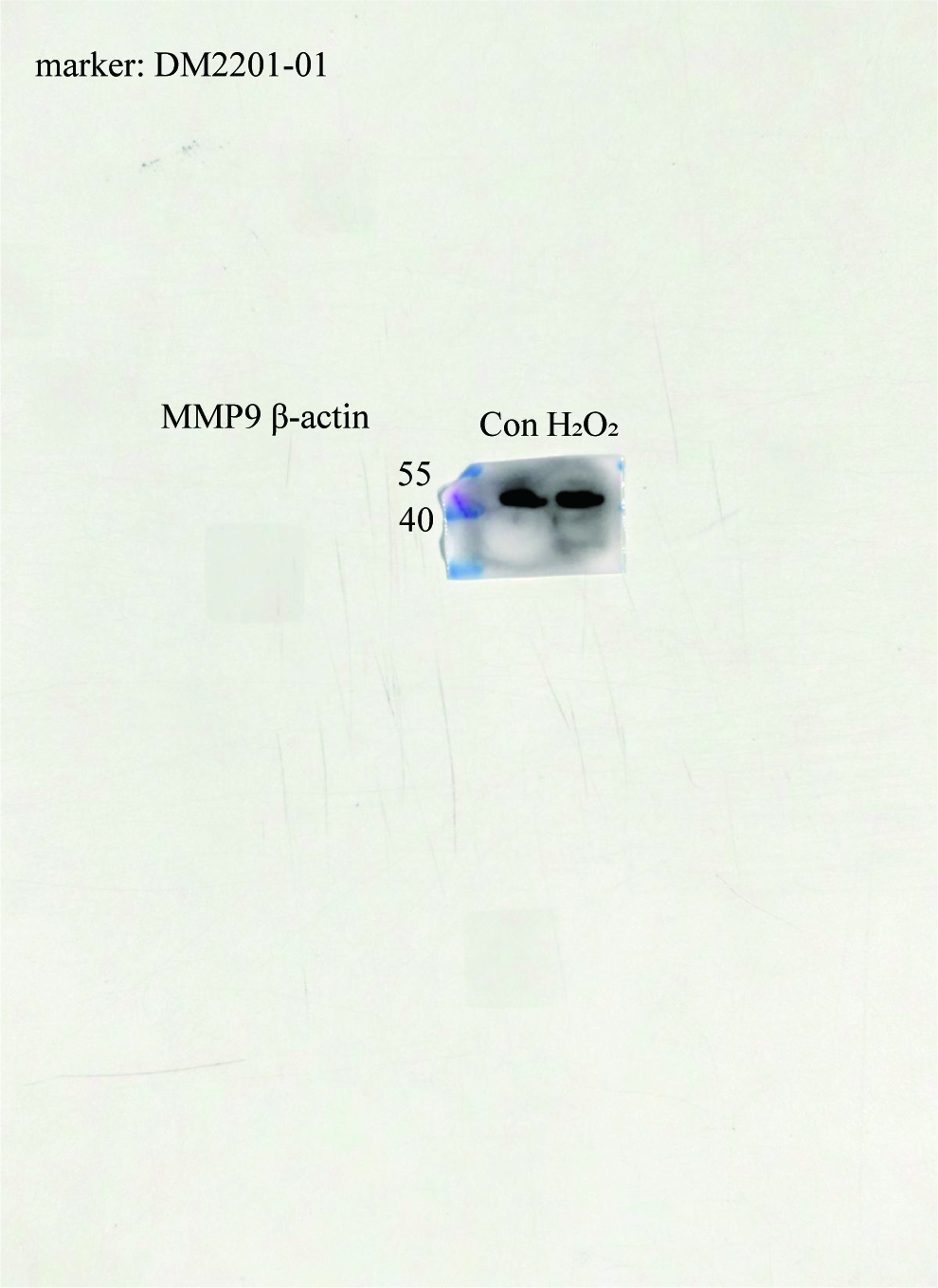

Supplement: Supplementary file 1 [file animals-15-01847-s001.zip › FIG. 5/MMP9 actin.tif]

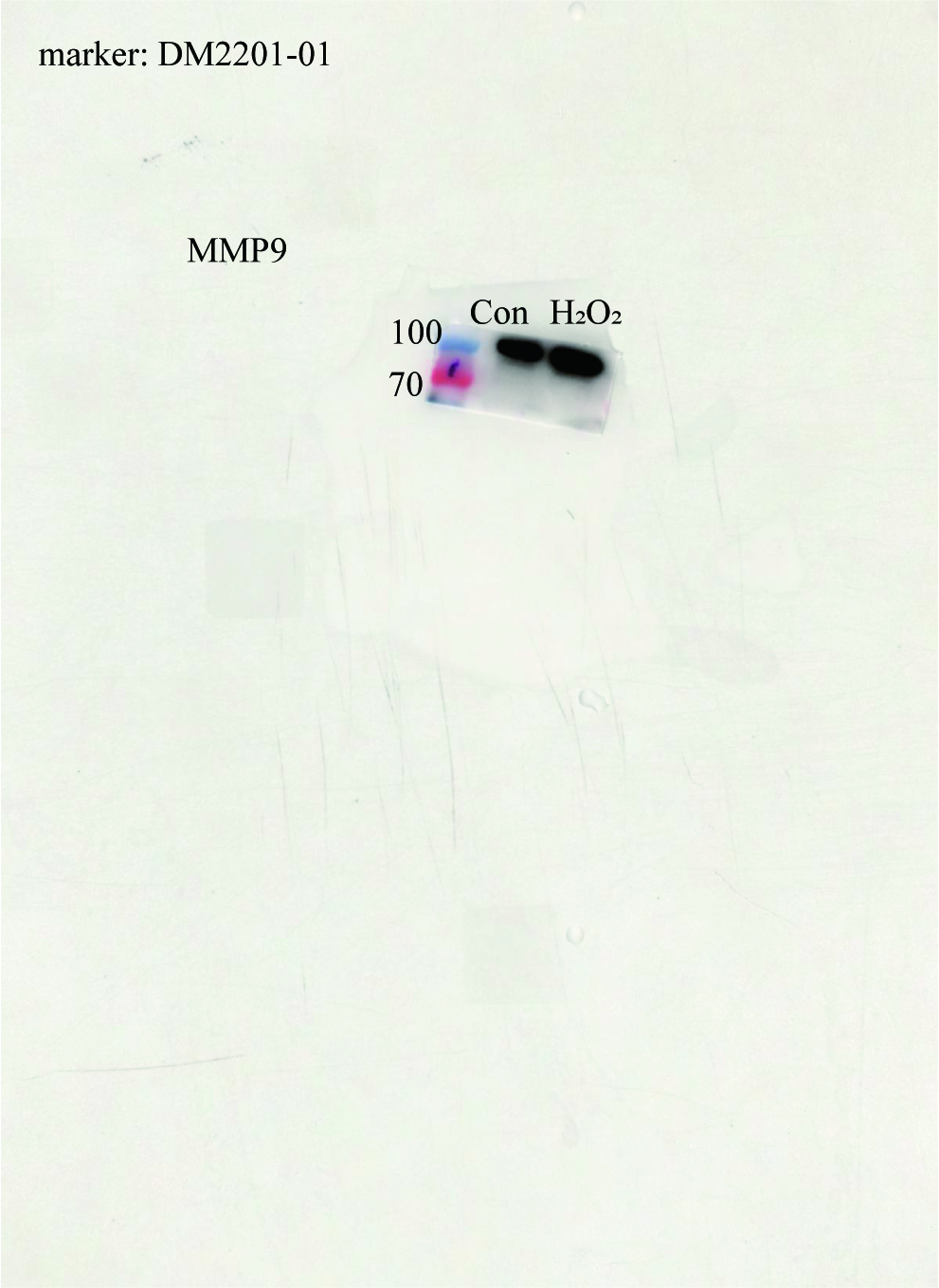

Supplement: Supplementary file 1 [file animals-15-01847-s001.zip › FIG. 5/MMP9.tif]

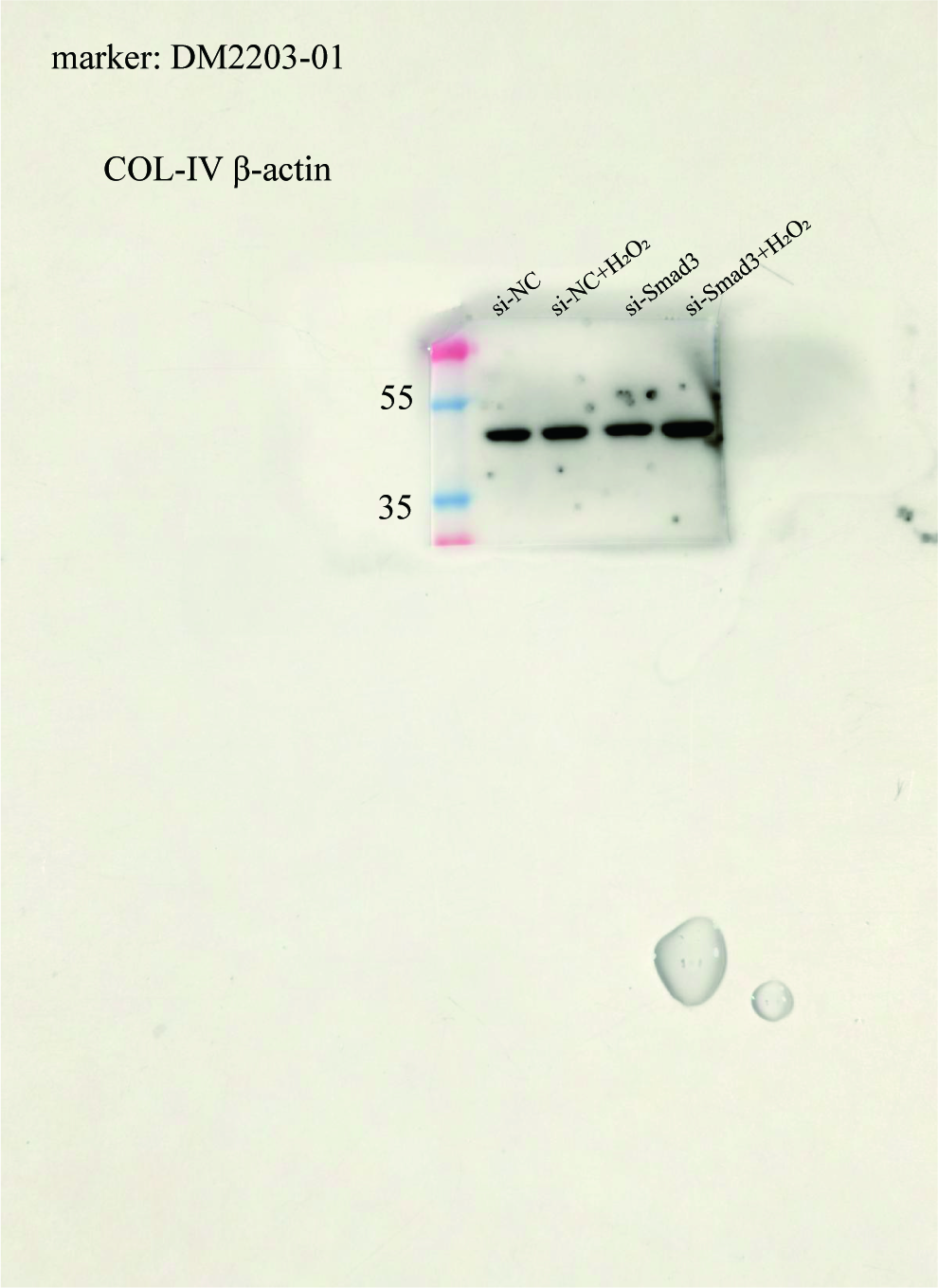

Supplement: Supplementary file 1 [file animals-15-01847-s001.zip › FIG. 6/COL-IV actin.tif]

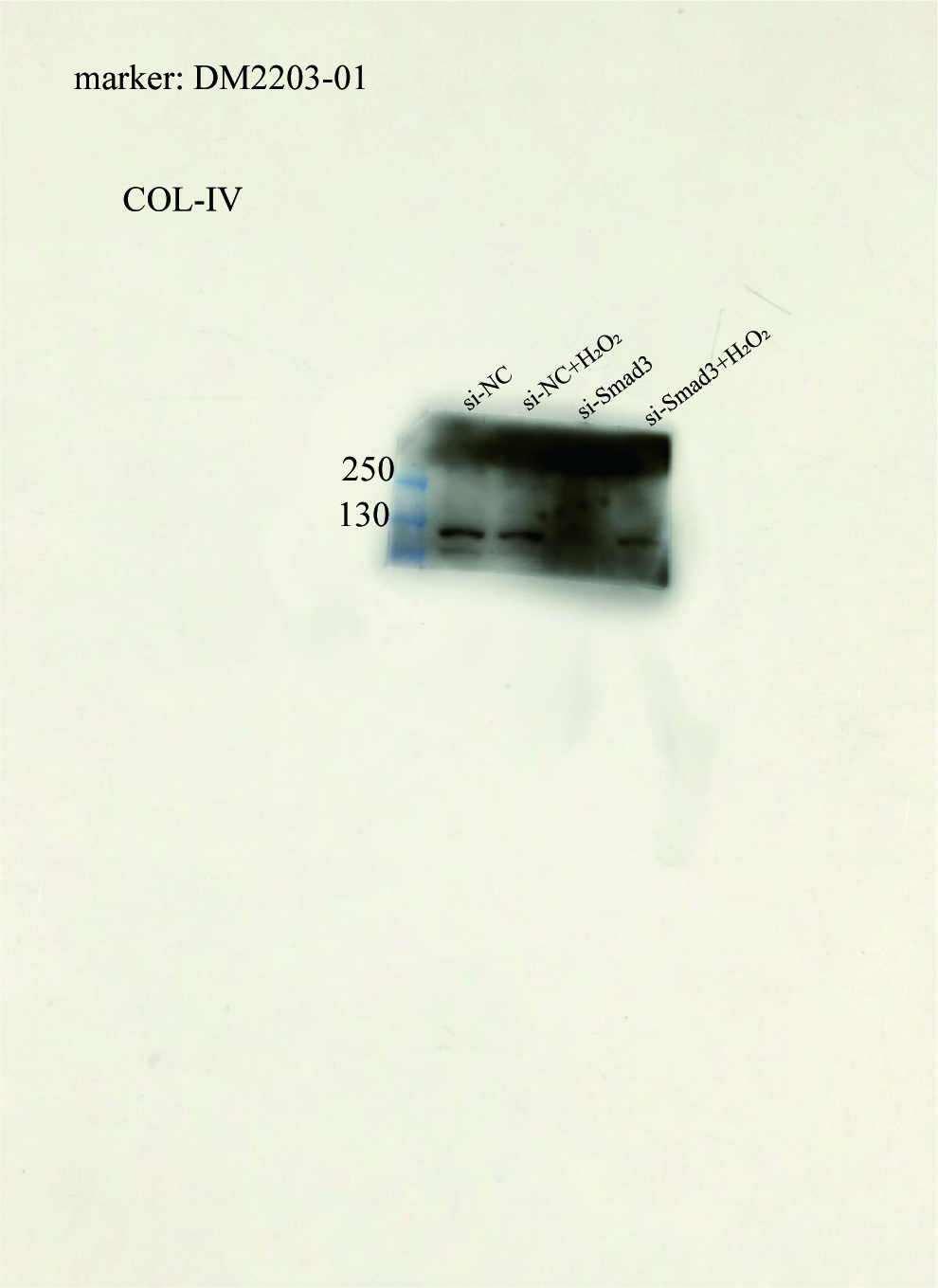

Supplement: Supplementary file 1 [file animals-15-01847-s001.zip › FIG. 6/COL-IV.tif]

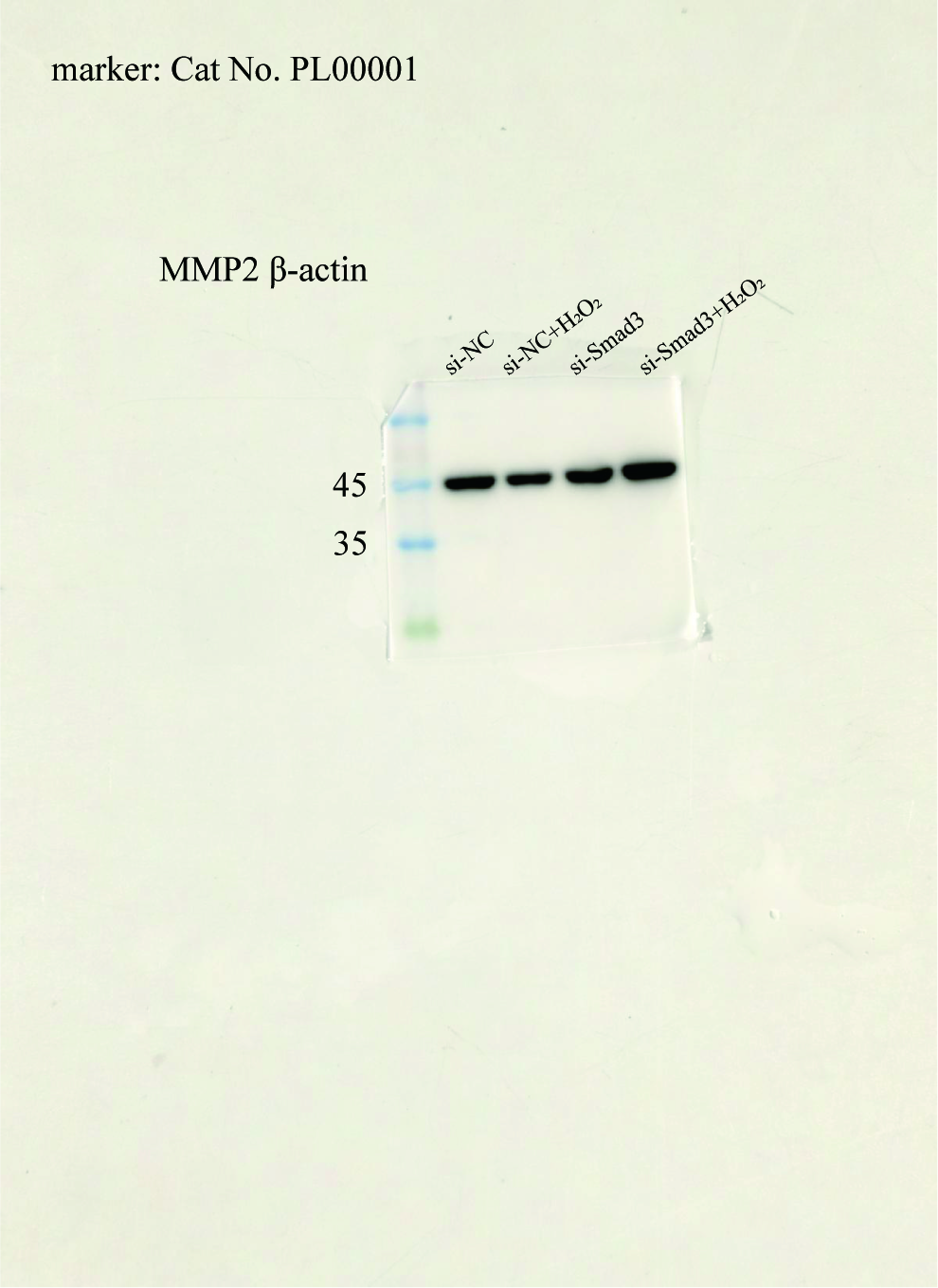

Supplement: Supplementary file 1 [file animals-15-01847-s001.zip › FIG. 6/MMP2 actin.tif]

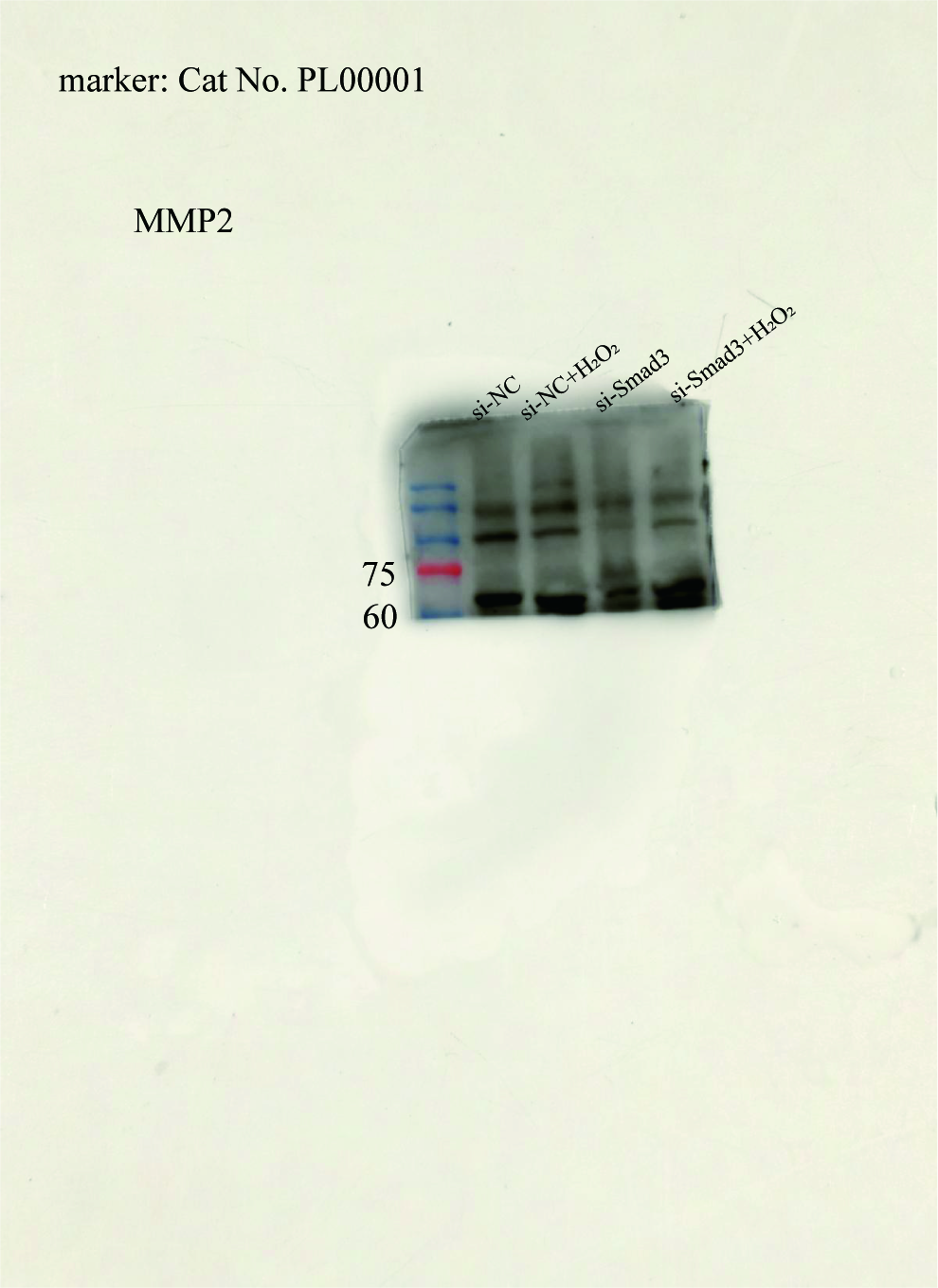

Supplement: Supplementary file 1 [file animals-15-01847-s001.zip › FIG. 6/MMP2.tif]

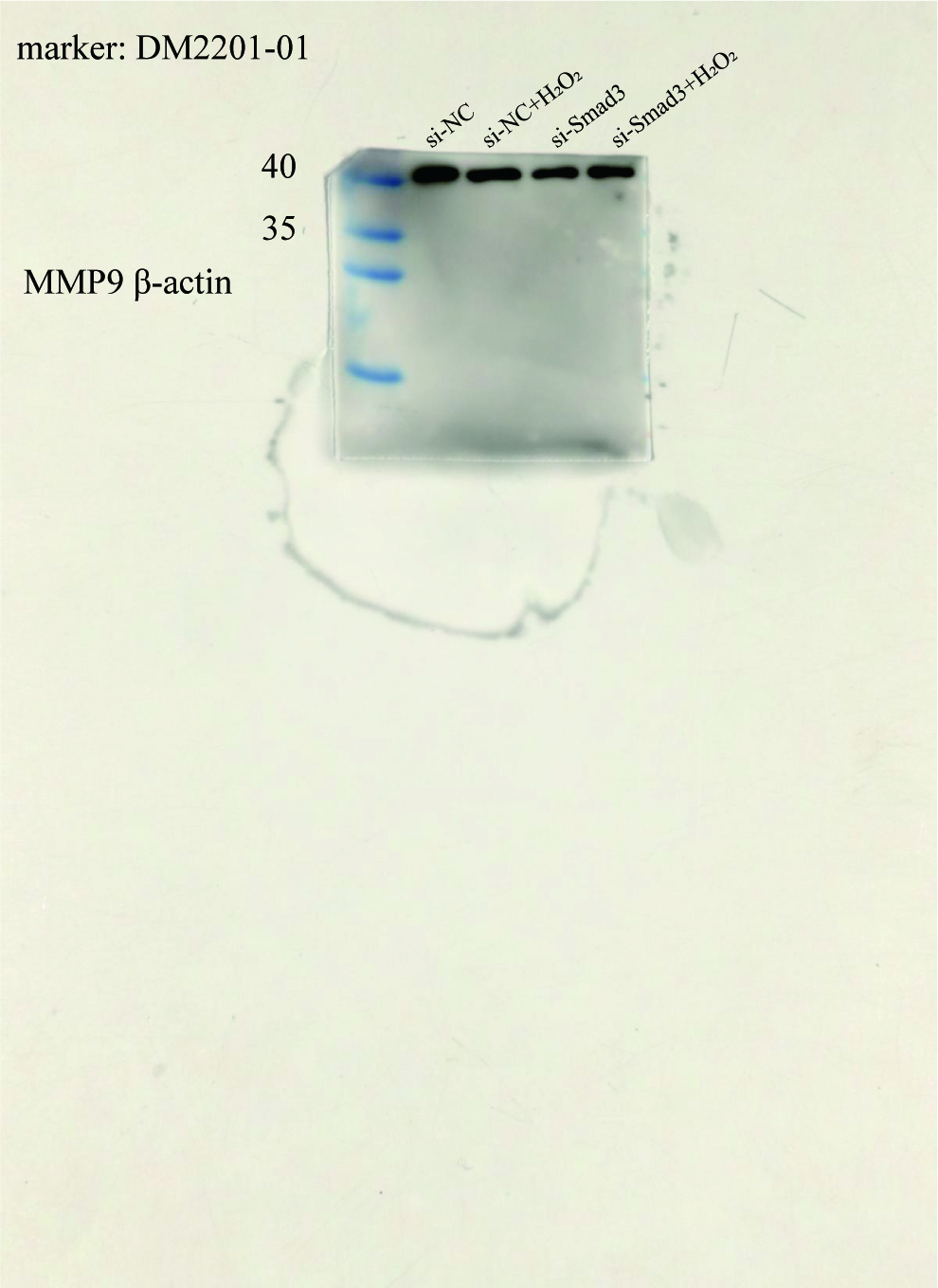

Supplement: Supplementary file 1 [file animals-15-01847-s001.zip › FIG. 6/MMP9 actin.tif]

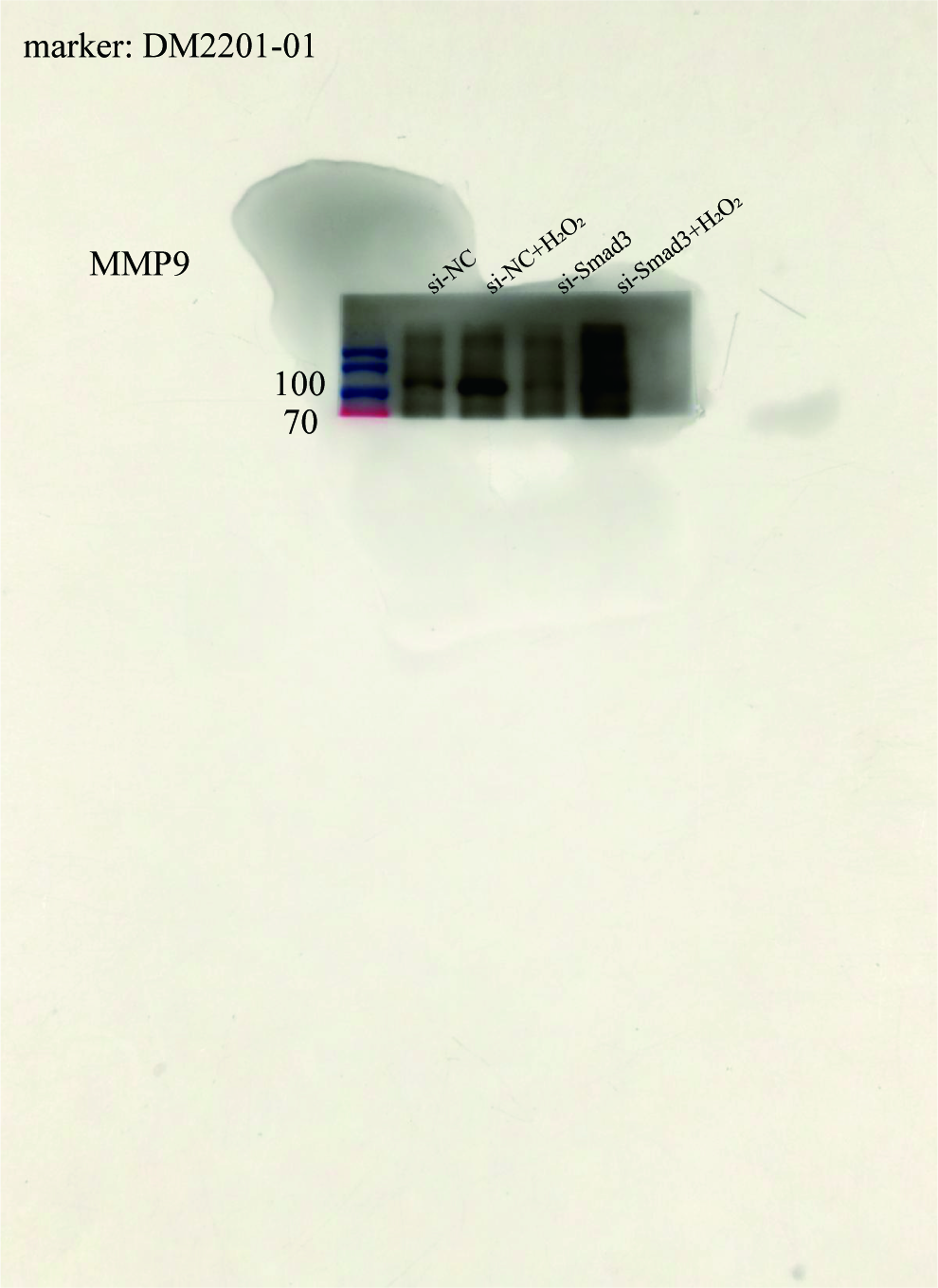

Supplement: Supplementary file 1 [file animals-15-01847-s001.zip › FIG. 6/MMP9.tif]

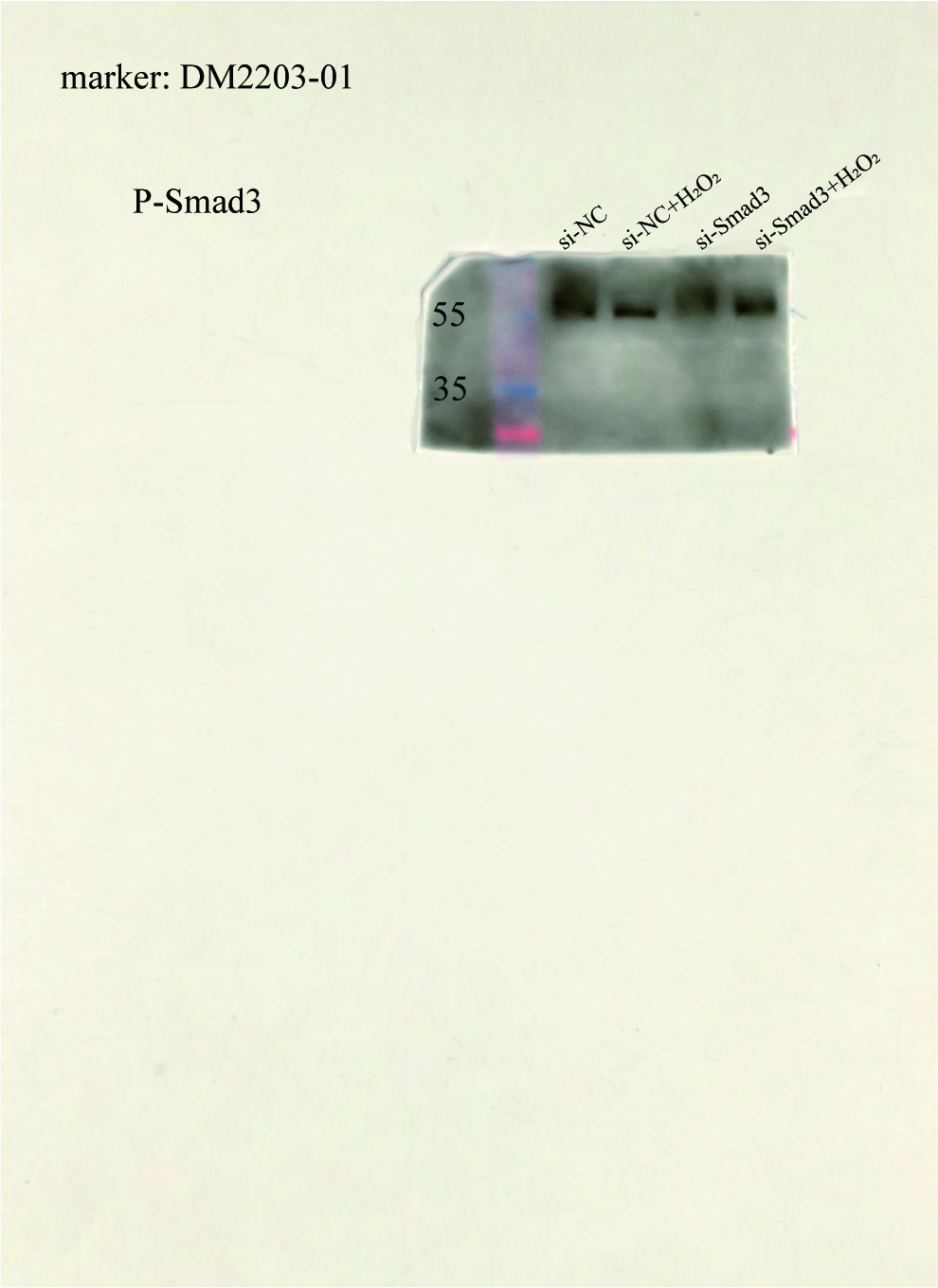

Supplement: Supplementary file 1 [file animals-15-01847-s001.zip › FIG. 6/P-smad3.tif]

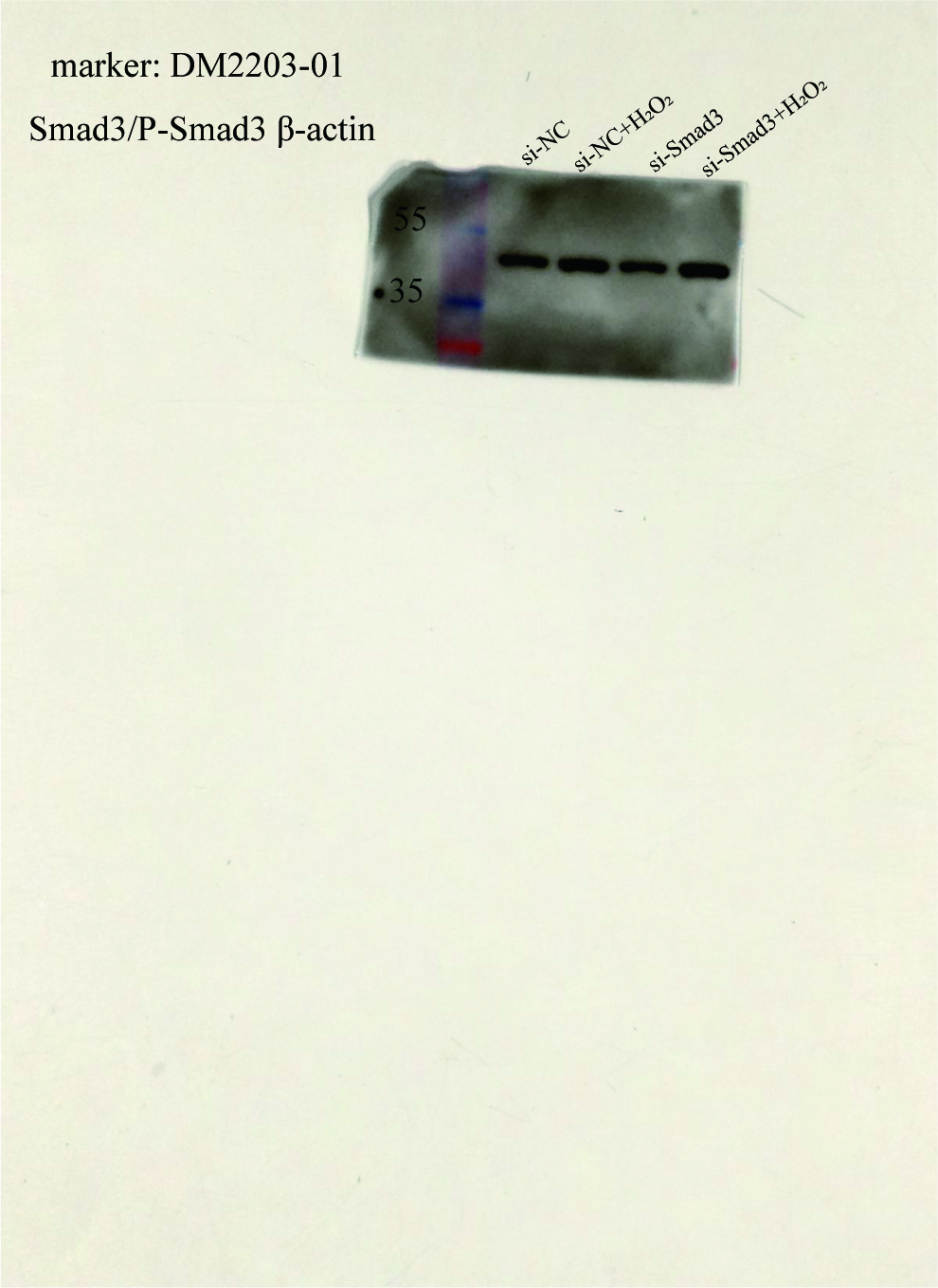

Supplement: Supplementary file 1 [file animals-15-01847-s001.zip › FIG. 6/smad3 actin.tif]

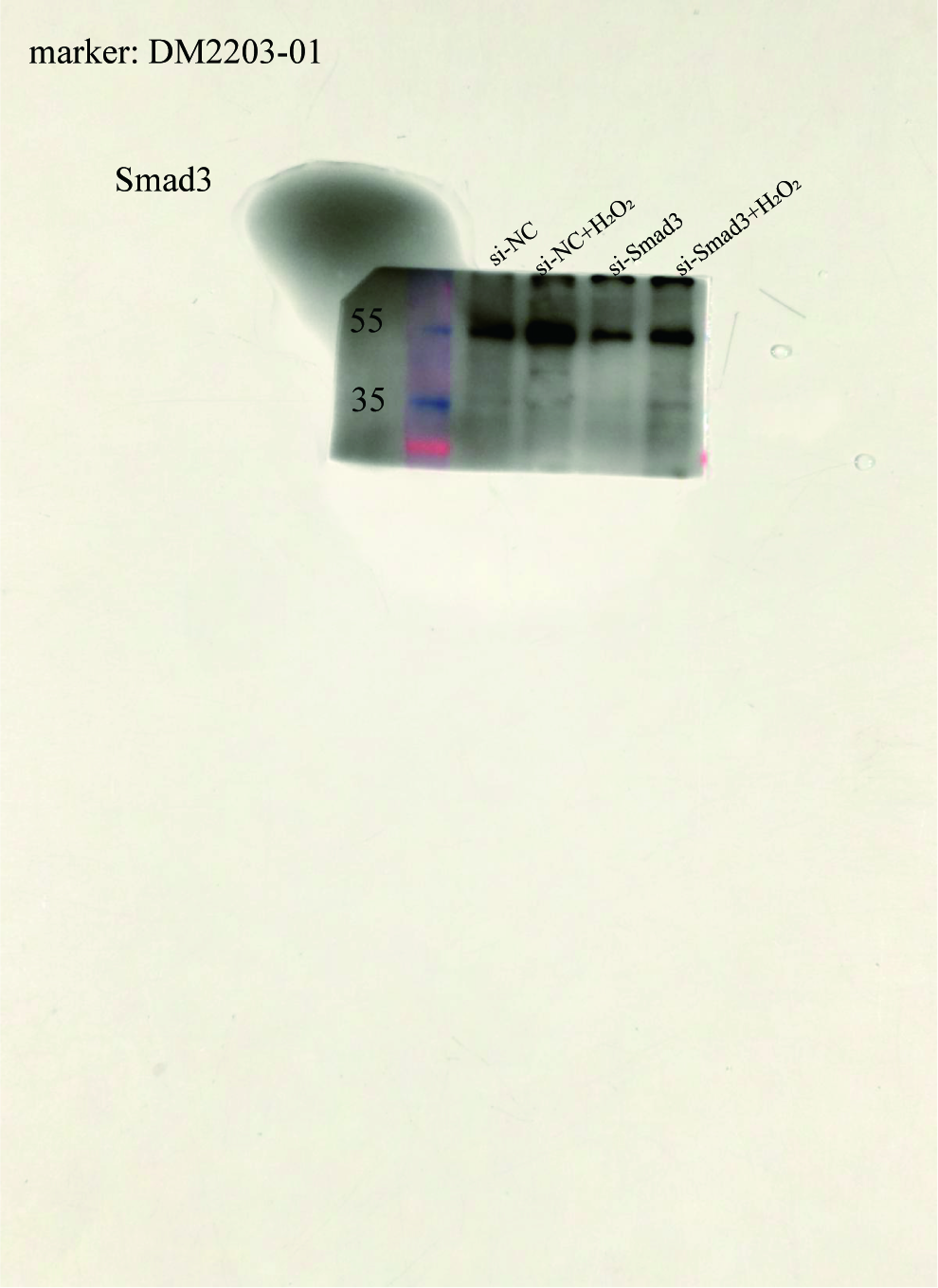

Supplement: Supplementary file 1 [file animals-15-01847-s001.zip › FIG. 6/smad3.tif]
